# A tactfully designed photothermal agent collaborating with ascorbic acid for boosting maxillofacial wound healing

**DOI:** 10.1093/nsr/nwae426

**Published:** 2024-11-26

**Authors:** Yuxin Qian, Yiting Gao, Dong Wang, Shixuan Zhang, Qiuxia Luo, Guogang Shan, Mengmeng Lu, Dingyuan Yan, Ben Zhong Tang, Ming Zhang

**Affiliations:** Department of Oral Implantology, The Affiliated Stomatological Hospital of Nanjing Medical University. State Key Laboratory Cultivation Base of Research, Prevention and Treatment for Oral Diseases. Jiangsu Province Engineering Research Center of Stomatological Translational Medicine, Nanjing Medical University, Nanjing 210029, China; Institute of Functional Material Chemistry and National & Local United Engineering Lab for Power Battery, Faculty of Chemistry, Northeast Normal University, Changchun 130024, China; Center for AIE Research, Guangdong Provincial Key Laboratory of New Energy Materials Service Safety, College of Materials Science and Engineering, Shenzhen University, Shenzhen 518060, China; Department of Oral Implantology, The Affiliated Stomatological Hospital of Nanjing Medical University. State Key Laboratory Cultivation Base of Research, Prevention and Treatment for Oral Diseases. Jiangsu Province Engineering Research Center of Stomatological Translational Medicine, Nanjing Medical University, Nanjing 210029, China; Center for AIE Research, Guangdong Provincial Key Laboratory of New Energy Materials Service Safety, College of Materials Science and Engineering, Shenzhen University, Shenzhen 518060, China; Institute of Functional Material Chemistry and National & Local United Engineering Lab for Power Battery, Faculty of Chemistry, Northeast Normal University, Changchun 130024, China; Department of Oral Implantology, The Affiliated Stomatological Hospital of Nanjing Medical University. State Key Laboratory Cultivation Base of Research, Prevention and Treatment for Oral Diseases. Jiangsu Province Engineering Research Center of Stomatological Translational Medicine, Nanjing Medical University, Nanjing 210029, China; Center for AIE Research, Guangdong Provincial Key Laboratory of New Energy Materials Service Safety, College of Materials Science and Engineering, Shenzhen University, Shenzhen 518060, China; Center for AIE Research, Guangdong Provincial Key Laboratory of New Energy Materials Service Safety, College of Materials Science and Engineering, Shenzhen University, Shenzhen 518060, China; School of Science and Engineering, Shenzhen Institute of Aggregate Science and Technology, The Chinese University of Hong Kong, Shenzhen (CUHK-Shenzhen), Shenzhen 518172, China; Department of Oral Implantology, The Affiliated Stomatological Hospital of Nanjing Medical University. State Key Laboratory Cultivation Base of Research, Prevention and Treatment for Oral Diseases. Jiangsu Province Engineering Research Center of Stomatological Translational Medicine, Nanjing Medical University, Nanjing 210029, China

**Keywords:** maxillofacial wound healing, photothermal sterilization, anti-inflammation

## Abstract

Maxillofacial injuries that may cause severe functional and aesthetic damage require effective and immediate management due to continuous exposure to diverse microbial populations. Moreover, drug resistance, biofilm formation, and oxidative stress significantly impede timely bacterial removal and immune function, making the exploration of advanced materials for maxillofacial wound healing an appealing yet highly challenging task. Herein, a near-infrared photothermal sterilization agent was designed, encapsulated with liposomes and coated with ascorbic acid known for its antioxidant and immune-regulatory functions. The resulting nanoparticles, 4TPE-C6T-TD@AA, effectively neutralize reactive oxygen species generated by lipopolysaccharides, facilitate the conversion of pro-inflammatory M1 macrophages to anti-inflammatory M2 macrophages, and eliminate >90% of *Staphylococcus aureus* and *Escherichia coli* by disrupting bacterial physiological functions upon exposure to 808 nm laser irradiation. *In vivo* experiments demonstrate that 4TPE-C6T-TD@AA rapidly eliminates bacteria from infected wounds in the maxillofacial region of rats, and significantly promotes healing in *S. aureus*-infected wounds by enhancing collagen formation and modulating the inflammatory microenvironment. In conclusion, this study presents a promising therapeutic strategy for effectively combating bacterial infections and excessive inflammation in treating maxillofacial injuries.

## Teaser

The study introduces a meticulously designed photothermal agent, named 4TPE-C6T-TD@AA, which enhances wound healing by eradicating bacteria, controlling inflammation and accelerating collagen synthesis in maxillofacial infected wounds.

## INTRODUCTION

The oral and maxillofacial region, often exposed to the external environment and characterized by complex anatomy, is highly susceptible to traumatic injuries resulting from falls or traffic accidents and subsequent bacterial infections [1]. If maxillofacial infections are not promptly managed, this region's function and aesthetics could be severely impaired. Therefore, managing infected wounds in the maxillofacial area is an appealing and significant challenge for clinicians [2,3]. The primary difficulty in treating these infections arises from their continuous exposure to diverse microbial populations. Bacteria colonize the wound, invade deep tissues, and secrete toxins that cause cellular destruction [4,5]. Moreover, biofilm formation on the wound surface protects bacteria, enabling them to proliferate and evade phagocytosis by immune cells [6]. Additionally, bacteria and their metabolites perpetuate the inflammatory response, prolonging the inflammatory phase of the wound, which delays collagen formation and repair [7]. Thus, there is an urgent need to develop effective antibacterial and anti-inflammatory strategies for treating infected wounds in the oral and maxillofacial region.

Bacterial infection represents a significant obstacle in wound treatment [8]. Antibiotics are commonly used in clinical settings for prevention and control [9]. However, antibiotic resistance has become a major global public health threat [10]. Consequently, as policies and procedures to limit antibiotic use are being formulated, researchers are exploring alternative treatment strategies [11–13]. Photothermal therapy (PTT) is a promising technology that uses photothermal agents (PTAs) to convert light energy into heat, generating local hyperthermia to destroy bacterial structures, eliminate bacteria, and facilitate wound healing [14,15]. Near-infrared (NIR, 700–1700 nm) light-induced PTT is highly suitable for clinical applications due to its reduced light damage to surrounding healthy tissues, deep penetration depth, and ability to simultaneously target multiple bacterial components, effectively avoiding bacterial resistance [16–18]. The choice of PTA is critical to the efficacy of PTT. Inorganic PTAs, such as metal nanoparticles (NPs), semiconductors, and carbon-based materials, usually possess poor biodegradability and potential long-term cumulative toxicity, hindering their clinical application [19,20]. In contrast, organic PTAs, which possess well-defined structures and are readily modified chemically, degrade and are excreted more efficiently, resulting in better biocompatibility and versatility [21,22]. To promote photothermal conversion, introducing sufficient molecular rotors is highly recommended [23–26]. The presence of molecular rotors facilitates active intramolecular motions due to their freely rotatable feature, and promotes a twisted molecular geometry that enables loose molecular packing, allowing PTT to be conducted effectively even in aggregated states. Therefore, constructing PTAs decorated with multiple molecular rotors represents an effective strategy for the rapid and efficient treatment of maxillofacial bacterial infections [27–29].

Wound healing is a multifaceted biological process that generally comprises the phases of hemostasis, inflammation, proliferation, and remodeling [30]. Efficient repair of damaged tissue requires the synergistic action and precise regulation of multiple cell types [31]. When bacteria and their metabolites invade wound tissues, they induce oxidative stress and produce large amounts of reactive oxygen species (ROS). This oxidative stress causes immune dysfunction, impairs inflammatory cell function, and prolongs the inflammatory phase of wound healing [32,33]. M1 pro-inflammatory macrophages accumulate in large numbers at the wound site and can quickly phagocytose bacteria in the short term [34]. However, prolonged infection and inflammation lead to a sustained increase in M1 macrophages, which delays wound healing [35]. To counteract ROS at the infection site, it is essential to introduce effective and safe antioxidants with high therapeutic value [36,37]. Ascorbic acid (AA) is a naturally occurring vitamin that has been shown to have potent antioxidant activity. Multiple clinical studies have shown that supplementing AA during infection and stress can effectively mitigate bacterial infections in the respiratory tract, digestive tract, and skin, as well as shorten treatment duration. For instance, Farhad *et al.* extracted AA from black pepper berries and formulated an ointment that accelerated wound healing and prevented scar formation when applied to wounds [38]. Additionally, AA is a cofactor for collagen hydroxylation and is necessary for collagen formation. Since collagen synthesis and accumulation are crucial for normal wound healing, an adequate supply of AA can significantly accelerate wound healing and regeneration [14,39].

**Scheme 1.**
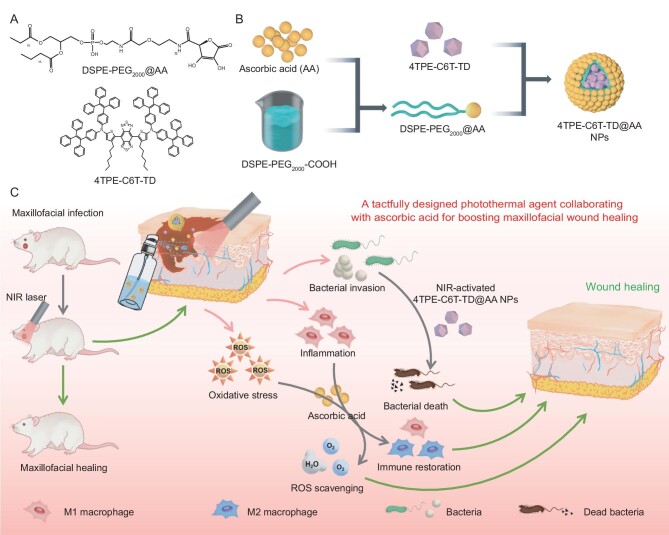
(A) Chemical structures of DSPE-PEG_2000_@AA and 4TPE-C6T-TD. (B) Flow chart illustrating the synthesis of 4TPE-C6T-TD@AA. (C) Schematic diagram depicting the application of 4TPE-C6T-TD@AA in treating maxillofacial infected wounds, promoting wound healing through its antibacterial, antioxidant, and anti-inflammatory effects.

Herein, we designed two NIR PTAs by incorporating multiple triphenylamine (TPA) or tetraphenylethylene (TPE) segments at the periphery of the molecules. To improve photothermal stability and conversion efficiency, *ortho*-alkylated thiophene was selected as a π-bridge to twist the molecular backbone. Additionally, the installation of four TPA or TPE moieties on the periphery of the molecule facilitates nonradiative decay. Considering that 4TPE-C6T-TD has a lower energy bandgap and higher light-capturing ability, which improve its photophysical properties, we selected this PTA for further investigation. Utilizing the drug-loading capacity of liposomes, we prepared the NPs by encapsulating 4TPE-C6T-TD and coating the surface with AA (Scheme 1). *In vitro* experiments verified the efficient photothermal bactericidal effect of the 4TPE-C6T-TD@AA NPs, along with their ability to scavenge ROS, regulate macrophage polarization, and modulate the immune microenvironment. *In vivo* experiments further confirmed their effectiveness in promoting rapid healing of maxillofacial infected wounds in rats.

## RESULTS AND DISCUSSION

### Molecular design and photophysical property

**Figure 1.**
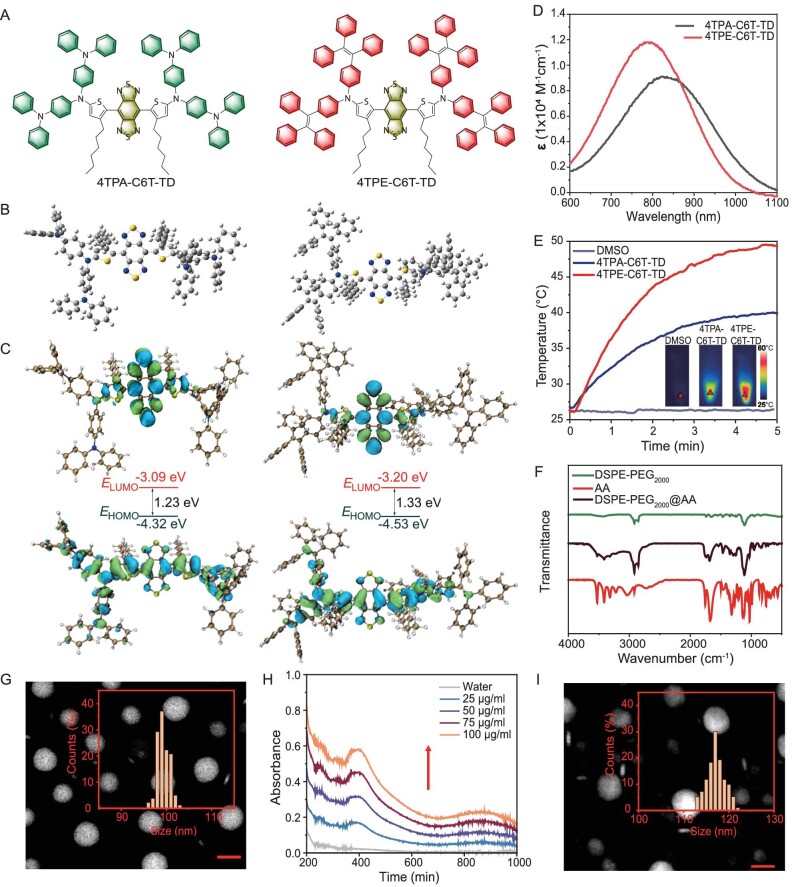
(A) Chemical structures of 4TPA-C6T-TD and 4TPE-C6T-TD. (B) Optimized S_0_ geometries of 4TPA-C6T-TD and 4TPE-C6T-TD. (C) Illustration of the frontier molecular orbitals (LUMOs and HOMOs) determined at the B3LYP/6–31G(d) level of theory. (D) The absorption spectra of obtained compounds (10 × 10^−6^ M) dissolved in THF solution. (E) Comparison of the photothermal conversion behavior of the two molecules (100 μM) in DMSO solution upon an 808 nm laser irradiation (1.0 W/cm^2^). (F) FTIR spectra of AA, DSPE- PEG_2000_, and DSPE- PEG_2000_@AA. (G) TEM image of 4TPE-C6T-TD@AA dispersed in water. Scale bar, 30 μm. (H) The absorption spectra of 4TPE-C6T-TD@AA at different concentrations. (I) TEM image and particle size analysis of 4TPE-C6T-TD@AA degraded in water for 7 days. Scale bar, 30 μm.

Benzobisthiadiazole (BBTD) is an optimal candidate for constructing NIR-absorbing molecules due to its strong electron-withdrawing capacity [40]. For twisting the molecular backbone, *ortho*-alkylated thiophene was selected as a π-bridge. Additionally, installing four TPA or TPE moieties at the molecular periphery enhances the donor–acceptor interactions and provides intense molecular rotors to facilitate nonradiative decay [29]. Accordingly, two molecules, 4TPA-C6T-TD and 4TPE-C6T-TD, were precisely designed and synthesized (Fig. 1A; Fig. S1). The structural characteristics of key intermediates and final products are detailed in Figs S2–S15. Density functional theory (DFT) calculations were carried out to investigate their structural features. The optimized ground-state (S_0_) geometries, shown in Fig. 1B, exhibit twisted conformations that prevent dense molecular packing in aggregates and promote intra- and intermolecular motions to generate heat. The frontier molecular orbitals of the two molecules were then determined based on the S_0_ geometries, revealing that the highest occupied molecular orbitals (HOMOs) are distributed along the entire molecular backbone, while the lowest unoccupied molecular orbitals (LUMOs) are mainly located on the BBTD unit. The energy bandgaps of 4TPA-C6T-TD and 4TPE-C6T-TD were calculated to be 1.23 and 1.33 eV, respectively (Fig. 1C). The photophysical properties of 4TPA-C6T-TD and 4TPE-C6T-TD were systematically investigated. As depicted in Fig. 1D, the absorption maxima of 4TPA-C6T-TD and 4TPE-C6T-TD in tetrahydrofuran (THF) solution were measured at 825 and 782 nm with molar extinction coefficients (ɛ) of 0.9 × 10^4^ and 1.15 × 10^4^ M^−1^ cm^−1^ at 808 nm, respectively. Due to its greater light-capturing ability, 4TPE-C6T-TD exhibited superior photothermal conversion efficiency under 808 nm laser irradiation in dimethyl sulfoxide (DMSO) solution, making it the preferred choice for the implementation of PTT (Fig. 1E).

### Synthesis and characterization of 4TPE-C6T-TD@AA

Liposomes have emerged as one of the most promising nanocarriers for local administration due to their excellent biocompatibility, biodegradability, and ability to accommodate hydrophilic drugs in internal water cavities and hydrophobic agents in bilayers [41]. To fabricate hydrophobic PTAs, water-dispersible AA was conjugated to 1,2-distearoyl-sn-glycero-3-phosphoethanolamine-*N*-[methoxy(polyethylene glycol)-2000] (DSPE-PEG_2000_). The successful synthesis of DSPE-PEG_2000_@AA was confirmed by FTIR spectra (Fig. 1F). 4TPE-C6T-TD@AA NPs were subsequently fabricated via an ultrasonic deposition. As shown in Fig. 1G, 4TPE-C6T-TD@AA NPs displayed a spherical liposome structure with a diameter of 177.5 nm, uniformly dispersed in water (Figs S16 and S17). The absorption spectra demonstrated that 4TPE-C6T-TD@AA exhibited increasing absorption peaks with rising concentration while maintaining the absorption maxima peak around 808 nm. This indicates that the optical properties of the 4TPE-C6T-TD remain unchanged upon encapsulation in liposomes (Fig. 1H). As shown in Fig. 1I, the NPs maintained their spherical morphology and consistent particle size after 7 days of dispersion in aqueous solution. This stability in water and suitability for storage indicates that 4TPE-C6T-TD@AA NPs can uniformly distribute on wound tissue, preventing local high-density aggregation.

**Figure 2.**
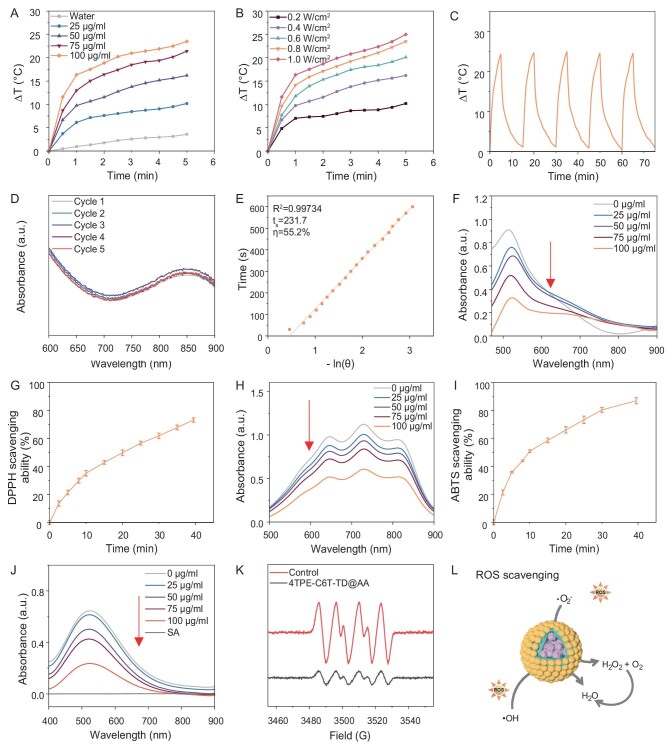
The photothermal and antioxidant properties of 4TPE-C6T-TD@AA. (A) Photothermal activity of 4TPE-C6T-TD@AA with different concentrations under 808 nm laser irradiation (1 W/cm^2^). (B) Photothermal activity of 4TPE-C6T-TD@AA (100 μg/mL) with different power densities of 808 nm laser irradiation. (C) Heating and cooling curves and (D) absorption spectra of the photothermal conversion cycling test of 4TPE-C6T-TD@AA. (E) Linear fitting of time versus -ln(*θ*) during cooling of 4TPE-C6T-TD@AA. (F) The absorption spectra and (G) kinetic curve analysis of the DPPH test evaluating the total ROS scavenging capacity of 4TPE-C6T-TD@AA. (H) The absorption spectra and (I) kinetic curve analysis of ABTS test evaluating the total ROS scavenging capacity of 4TPE-C6T-TD@AA. (J) Absorption spectra of Fenton reaction examining the ·OH scavenging capacity of 4TPE-C6T-TD@AA. (K) Electron spin resonance (ESR) measurement of ·O^2−^ scavenging capacity of 4TPE-C6T-TD@AA. (L) Proposed mechanism of 4TPE-C6T-TD@AA to scavenge ROS (*n =* 3). Statistical analysis was performed using one-way ANOVA with Tukey's post-test. *p < 0.05, **p < 0.01, ***p < 0.001.

An 808 nm laser was selected as the emission source to evaluate the photothermal performance of 4TPE-C6T-TD@AA. Infrared thermal images revealed that the photothermal conversion of 4TPE-C6T-TD@AA aqueous solution depended on both concentration and power density (Figs S18 and S19). At a power density of 1.0 W/cm^2^, the temperature of 4TPE-C6T-TD@AA aqueous solution (100 μg/ml) rapidly rose to 25.0°C (Fig. 2A and B). To assess the photothermal stability of 4TPE-C6T-TD@AA, indocyanine green (ICG), the only FDA-approved photothermal transduction agent, was used as a control in the photothermal cycling experiment. Following five cycles, the photothermal efficacy of ICG gradually diminished, concomitant with notable changes in solution color after the second cycle (Fig. 2C and D; Fig. S20). In contrast, 4TPE-C6T-TD@AA exhibited a consistent maximum temperature throughout the cycling regimen, underscoring its exceptional thermal stability (Figs S21–S23). This stability prevents unnecessary material inactivation, allowing multiple efficient PTT treatments within a single course of therapy. As shown in Fig. 2E, the photothermal conversion efficiency (PCE) of 4TPE-C6T-TD@AA NPs was determined to be 55.2%, indicating the significant potential for photothermal sterilization.

Antioxidants can neutralize excessive ROS, thereby reversing the inflammatory microenvironment of wounds and promoting healing [42,43]. We initially assessed the antioxidant properties of 4TPE-C6T-TD@AA by measuring its ROS scavenging ability in solution. ABTS^+^ and DPPH^+^ radicals were generated by pre-oxidation and can be neutralized by antioxidants (Fig. S24). Both assays detected the concentration-dependent ability of 4TPE-C6T-TD@AA to scavenge total ROS in solution. At a concentration of 100 μg/mL, NPs demonstrated the capacity to neutralize 76.7% of DPPH and 90.1% of ABTS radicals. Kinetic analyses unveiled a prompt onset of ROS scavenging activity at the onset of the reaction, progressively eradicating residual ROS over time. This antioxidative process accelerated ROS neutralization within wound tissues, thereby mitigating oxidative stress, sustaining wound stability, promoting tissue regeneration, reducing infection susceptibility, and expediting wound healing upon localized application of 4TPE-C6T-TD@AA (Fig. 2F–I; Fig. S25). Based on the chemical interaction between ascorbic acid and hydroxyl radicals (·OH), we investigated the ability of the NPs to scavenge ·OH (Fig. 2J). This investigation revealed its significant efficacy in clearing ·OH (Fig. S26). Additionally, electron paramagnetic resonance (EPR) analysis demonstrated the proficiency of 4TPE-C6T-TD@AA in scavenging superoxide anion radicals (·O^2−^), as indicated by a marked reduction in the ·O^2−^ radical signal within the spectrum (Fig. 2K). These findings underscored the significant proficiency of 4TPE-C6T-TD@AA in mitigating various types of ROS (Fig. 2L).

### Antibacterial performance of 4TPE-C6T-TD@AA

**Figure 3.**
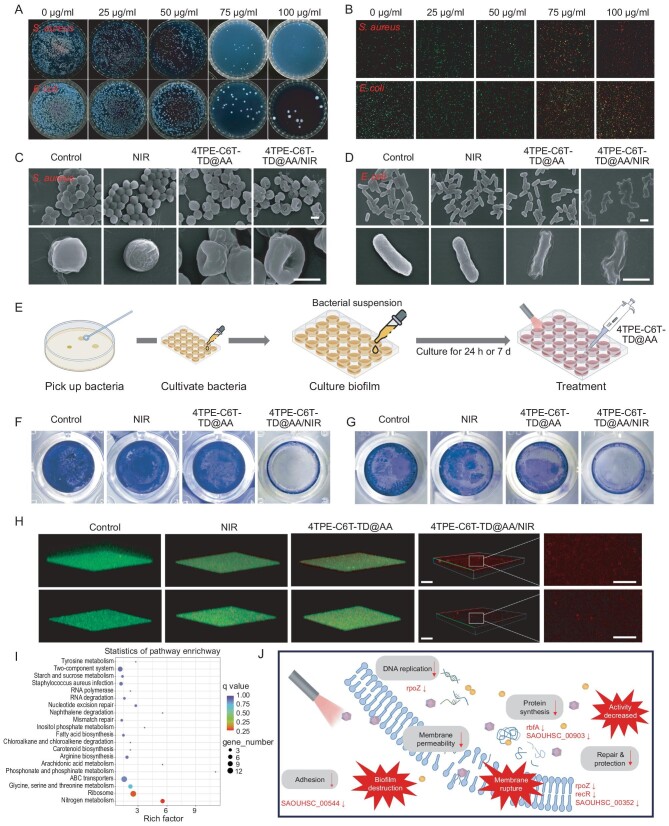
*In vitro* antibacterial behavior of 4TPE-C6T-TD@AA. (A) Typical photographs of bacterial colonies on agar plates and (B) Live/Dead staining images of *S. aureus* and *E. coli* after treatment with different concentrations of 4TPE-C6T-TD@AA under 808 nm laser irradiation. Scale bar, 100 μm. (C) Representative SEM images of bacterial morphologies of *S. aureus*. Scale bar, 500 nm. (D) Representative SEM images of bacterial morphologies of *E. coli*. Scale bar, 1 μm. (E) Schematic diagram of biofilm culture. Crystal violet staining images of 7-day (F) *S. aureus* and (G) *E. coli* biofilms. (H) 3D confocal Live/Dead staining images of 7-day biofilms. Scale bar, 100 μm. (I) KEGG enrichment analysis of differentially expressed genes (DEGs) in *S. aureus* after being treated with 4TPE-C6T-TD@AA/NIR compared to an untreated control. (J) Schematic diagram of the mechanism of 4TPE-C6T-TD@AA killing *S. aureus* with NIR (*n =* 3). Statistical analysis was performed using one-way ANOVA with Tukey's post-test. *p < 0.05, **p < 0.01, ***p < 0.001.

Maxillofacial wounds are highly susceptible to bacterial infections, leading to symptoms such as pain, swelling, and fever, which may result in severe complications [44]. We evaluated the antibacterial ability of 4TPE-C6T-TD@AA using colony-forming unit (CFU) assay and bacterial Live/Dead staining. As shown in Fig. 3A, the efficient photothermal conversion efficiency of 4TPE-C6T-TD@AA NPs led to a significant reduction in bacterial colonies on agar plates which was observed when the concentration of 4TPE-C6T-TD@AA NPs exceeded 50 μg/mL. At 100 μg/mL, only a few colonies remained on the plates. Bacterial Live/Dead staining images further visualized the antibacterial effect across different groups, consistent with the plate experiment results. Live bacteria stained with SYTO-9 exhibited green fluorescence, while dead bacteria were labeled with red propidium iodide (PI). Bacteria primarily emitted green fluorescence with pure NIR irradiation, while the higher concentration of 4TPE-C6T-TD@AA resulted in increased red fluorescence under NIR irradiation. In the 100 μg/mL 4TPE-C6T-TD@AA treatment group, almost all bacteria were red (Fig. 3B). Quantitative analysis of the antibacterial effect was based on results from the spread plate technique. Following 5 min of 808 nm laser irradiation, 4TPE-C6T-TD@AA aqueous solution at 100 μg/mL eradicated over 90% of both *Staphylococcus aureus* and *Escherichia coli* (Fig. S27). It has been demonstrated that PTT can disrupt membrane integrity, resulting in the leakage of ions and proteins from the cytoplasm [16]. SEM observations confirmed this concept. Bacteria in the control group exhibited typical spherical or rod-shaped morphology with smooth and intact cell membranes for both *S. aureus* and *E. coli*. In contrast, bacteria in the NIR group displayed roughened surfaces, those in the 4TPE-C6T-TD@AA group exhibited distorted morphology, and the 4TPE-C6T-TD@AA/NIR group showed significant membrane rupture, indicating a notable decrease in bacterial viability (Fig. 3C and D). Supplementary experiments on the survival of bacteria treated with the NPs alone revealed that 4TPE-C6T-TD@AA itself exerted an inhibitory effect on bacterial growth, with NIR acting as a potent enhancer, amplifying its antibacterial activity (Figs S28–S30).

Biofilms protect bacteria through extracellular polymeric substances, consequently enhancing their resistance to antibiotics [45,46]. To evaluate the inhibitory effect of 4TPE-C6T-TD@AA/NIR on biofilm formation, we examined both newly formed biofilms, cultured for 24 hours and mature biofilms cultured for 7 days. This approach enabled us to assess the treatment's efficacy in inhibiting biofilm formation and eradicating established biofilms, providing insights into its therapeutic potential (Fig. 3E). Crystal violet staining was initially employed to visualize and quantify the biofilms of *S. aureus* and *E. coli*. Newly formed biofilms appeared as thin layers of purple after 24 hours, while mature biofilms, cultured for 7 days, appeared dense and deeply stained. Treatment with light alone or NPs alone did not significantly alter staining. However, when combined with NIR laser irradiation, 4TPE-C6T-TD@AA effectively removed biofilms of both *S. aureus* and *E. coli* (Fig. 3F and G; Fig. S31). The OD values of crystal violet stained biofilms were measured to quantify the remaining biofilm content (Figs S32 and S33). After treatment with 4TPE-C6T-TD@AA/NIR, residual biofilm content decreased to 1.5% for *S. aureus* and 1.9% for *E. coli*, indicating high efficacy. 3D fluorescence images of *S. aureus* and *E. coli* biofilms were then captured using a confocal laser scanning microscope (CLSM). Live/Dead staining was used to dye living bacteria green and dead bacteria red (Fig. 3H; Fig. S34). Consistent with crystal violet staining results, bacterial biofilms in the control group predominantly displayed green-stained live bacteria. Conversely, the NIR and 4TPE-C6T-TD@AA groups showed fewer live bacteria, with a minor presence of red-fluorescent dead bacteria.

Notably, the 4TPE-C6T-TD@AA/NIR group exhibited a significant population of dead bacteria, indicated by red fluorescence, in both 24-hour and 7-day biofilms. Moreover, the cross-sectional analysis showed that the thickness of the biofilm was significantly reduced under this treatment strategy (Fig. S35). These findings strongly suggested that when combined with photothermal effects, 4TPE-C6T-TD@AA not only partially inhibits biofilm formation but also effectively eliminates live bacteria within the biofilm, thereby eradicating *S. aureus* and *E. coli* biofilms.

To elucidate the potential synergistic antibacterial mechanism of 4TPE-C6T-TD@AA, RNA sequencing (RNA-seq) was conducted to analyze the gene expression profiles of bacteria in control, 4TPE-C6T-TD@AA, and 4TPE-C6T-TD@AA/NIR groups. Differential expression genes (DEGs) among the groups were visualized with a Venn diagram (Fig. S36). Subsequent volcano plot analysis unveiled that, compared to the control group, the 4TPE-C6T-TD@AA group manifested 547 upregulated and 571 downregulated genes, while the 4TPE-C6T-TD@AA/NIR group exhibited 529 upregulated and 501 downregulated genes. Furthermore, in comparison with the 4TPE-C6T-TD@AA group, the 4TPE-C6T-TD@AA/NIR group showcased 82 upregulated and 97 downregulated genes, signifying a substantial impact of both 4TPE-C6T-TD@AA and 4TPE-C6T-TD@AA/NIR on *S. aureus* (Fig. S37). Gene Ontology (GO) enrichment analysis indicated that gene alterations in the 4TPE-C6T-TD@AA and 4TPE-C6T-TD@AA/NIR groups were intricately linked to various biological processes, cellular components, and molecular functions (Fig. S38).

Moreover, the *Kyoto Encyclopedia of Genes and Genomes* (KEGG) pathway enrichment analysis showed that the differential genes between the 4TPE-C6T-TD@AA group and the control group were enriched in pathways related to sugar metabolism, bacterial infection, invasion, and DNA replication. These pathways are crucial for bacterial proliferation and virulence. Similarly, differential genes between the 4TPE-C6T-TD@AA/NIR group and the control group were enriched in pathways linked to sugar metabolism, amino acid metabolism and synthesis, monobactam biosynthesis, terpenoid backbone biosynthesis, and ribosomes—pathways vital for bacterial growth and proliferation.

Notably, compared to the 4TPE-C6T-TD@AA group, the 4TPE-C6T-TD@AA/NIR group exhibited significant differences in genes associated with sugar, amino acid, and fatty acid metabolism pathways, and additionally showed downregulation in genes related to RNA polymerase, RNA degradation, DNA repair, and nucleotide excision repair (Fig. 3I; Fig. S39), underscoring a more pronounced disruptive effect of 4TPE-C6T-TD@AA/NIR on the genetic material of *S. aureus*. Compared to the control group, the 4TPE-C6T-TD@AA/NIR group showed differential gene enrichment in key processes essential for genetic expression, including translation, ribosome assembly, transcription, DNA template regulation, and macromolecule biosynthesis. Notably, genes encoding bacterial DNA replication (e.g. *rpoZ, dnaA*, and *SAOUHSC_01 095*) and repair (e.g. *recA, recR*, and *hslO*) were markedly downregulated in the 4TPE-C6T-TD@AA/NIR group, indicating early-stage interference with gene expression (Fig. S40). Particularly, the downregulation of heat shock protein 33 (HSP33) encoded by *hslO* diminished the bacteria's stress resistance, expediting the process of bacterial thermal damage. Key genes implicated in transcription and translation of ribosomes (e.g. *rpsU, rpmH*, and *rplT*) were also significantly downregulated, indicative of further disruption by 4TPE-C6T-TD@AA/NIR in vital physiological processes such as peptide and protein synthesis. The downregulation of genes closely associated with biological macromolecules such as peptide folding and synthesis of bioactive proteins (e.g. *SAOUHSC_01 182, SAOUHSC_01 058*, and *SAOUHSC_00 903*) suggested disturbance even in the final step of protein production in genetic information expression in *S. aureus*. These findings underscored the close relationship between bacterial demise and inhibition or interference with multiple processes in DNA expression. qRT-PCR was used to verify changes in these markers at the mRNA level (Fig. S41). Through multi-level destructive effects, 4TPE-C6T-TD@AA/NIR efficaciously inhibited bacterial proliferation and survival (Fig. 3J).

### Reversing inflammatory microenvironment ability evaluation of 4TPE-C6T-TD@AA

To assess the *in vitro* biocompatibility of 4TPE-C6T-TD@AA, Cell Counting Kit-8 (CCK8) assays and Live/Dead staining experiments were conducted. Following co-culture with varying concentrations of 4TPE-C6T-TD@AA, the viability of L929 fibroblast cells and human oral keratinocytes (HOK) remained consistently high. Live/Dead fluorescence staining further revealed predominantly green staining of cells, with only sporadic instances of red fluorescence, indicating the high biocompatibility of 4TPE-C6T-TD@AA with the cells (Figs S42 and S43). To access the *in vivo* toxicity of 4TPE-C6T-TD@AA, organs of mice from both the PBS control group and the 4TPE-C6T-TD@AA experimental group, following tail vein injection, were subjected to the hematoxylin and eosin (H&E) staining. H&E staining showed no discernible pathological alterations in the heart, liver, spleen, lungs, and kidneys of mice in the 4TPE-C6T-TD@AA experimental group compared to the PBS control group (Fig. S44). This observation suggests that the degradation products of 4TPE-C6T-TD@AA do not induce damage to the internal organs of mice, thereby affirming the favorable biological safety profile of 4TPE-C6T-TD@AA.

**Figure 4.**
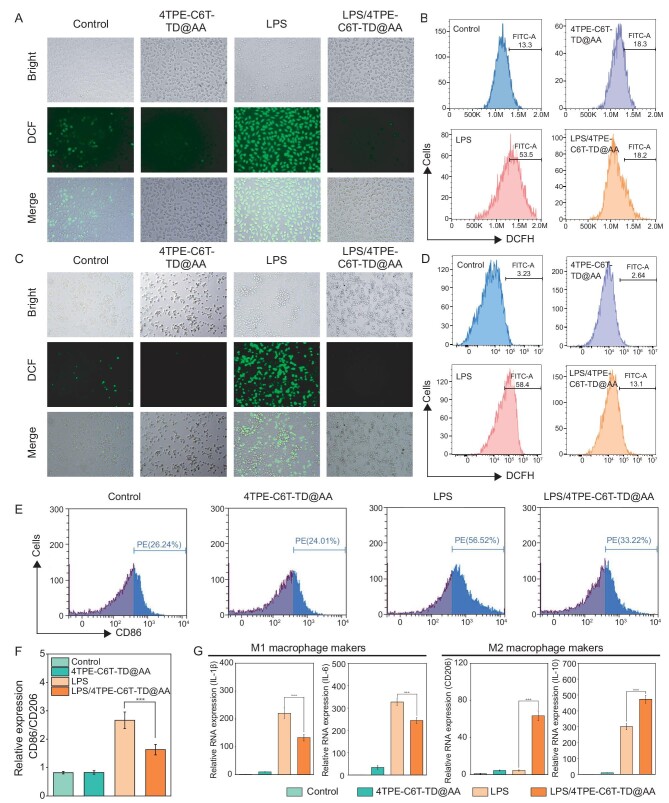
*In vitro* anti-inflammatory properties of 4TPE-C6T-TD@AA. (A) Light microscopic and DCFH-DA fluorescence images of RAW 264.7 macrophages. Scale bar, 100 μm. (B) Flow cytometry analysis of DCFH-DA expression in RAW 264.7 macrophages. (C) Light microscopic and DCFH-DA fluorescence images of L929 cells. Scale bar, 100 μm. (D) Flow cytometry analysis of DCFH-DA expression in L929 cells. (E) Flow cytometry assessment of CD86 expression, a macrophage marker, in RAW 264.7 macrophages. (F) Quantitative analysis of CD86/CD206 ratio in RAW 264.7 macrophages. (G) Quantitative analysis of immune factor expression in RAW 264.7 macrophages using qRT-PCR (*n* = 3). Statistical analysis was performed using one-way ANOVA with Tukey's post-test. *p < 0.05, **p < 0.01, and ***p < 0.001.

To evaluate the role of 4TPE-C6T-TD@AA in regulating the inflammatory microenvironment, an inflammatory environment was simulated by adding lipopolysaccharide (LPS) from *E. coli* to the culture medium. LPS stimulation significantly increased cellular ROS levels, leading to an excessive inflammatory response. 2′,7′-Dichlorodihydrofluorescein diacetate (DCFH-DA) probe was utilized to detect ROS, as ROS oxidizes non-fluorescent DCFH to produce green fluorescent DCF. The investigations of intracellular ROS clearance following a 24-hour treatment with 4TPE-C6T-TD@AA revealed that the control group exhibited minimal green fluorescence, while LPS-induced oxidative stress caused a notable increase in intracellular green fluorescence (Fig. 4A). In stark contrast, both the 4TPE-C6T-TD@AA and 4TPE-C6T-TD@AA/NIR groups displayed virtually no green fluorescence. Those results indicated that 4TPE-C6T-TD@AA effectively clears ROS in RAW 264.7 macrophages, regardless of whether ROS was induced by LPS. Additionally, flow cytometry analysis, as shown in Fig. 4B, corroborated the ROS clearance within RAW 264.7 macrophages by 4TPE-C6T-TD@AA. Compared to the LPS group (53.5% and 58.4%, respectively), 4TPE-C6T-TD@AA significantly reduced ROS production induced by LPS (18.2% and 13.1%, respectively). A comparative experiment with pure AA confirmed its role as an effective antioxidant in 4TPE-C6T-TD@AA for scavenging free radicals (Figs S45 and S46). Similarly, intracellular ROS clearance by 4TPE-C6T-TD@AA in L929 cells yielded similar results (Fig. 4C and D).

Simultaneously, light microscope images clearly showed that macrophages stimulated by LPS exhibited a flattened and slender form with numerous pseudopodia, characteristic of M1 macrophages. However, after co-culturing with 4TPE-C6T-TD@AA, the morphology of stimulated macrophages changed significantly, becoming rounder with shorter pseudopodia, resembling M2 macrophages [47]. To evaluate the influence of 4TPE-C6T-TD@AA on the polarization state of RAW 264.7 macrophages, flow cytometry was performed to detect CD86 and CD206 expression under LPS-induced inflammatory conditions. Compared to the control group, there was a significant increase in CD206 expression and a decrease in CD86 expression in cells treated with 4TPE-C6T-TD@AA (Fig. 4E and F). Since CD86 and CD206 are classical markers of M1 and M2 macrophages, these results demonstrate that 4TPE-C6T-TD@AA can remedy macrophages polarized from the M1 type guided by local infection and oxidative stress to the M2 type, which represents the anti-inflammatory type. Moreover, qRT-PCR analysis of related mRNA confirmed the flow cytometry results (Fig. 4G). These findings preliminarily suggest that 4TPE-C6T-TD@AA effectively promotes immune modulation towards an anti-inflammatory phenotype.

### Evaluation of the effect of 4TPE-C6T-TD@AA on the wound model of maxillofacial infection

**Figure 5.**
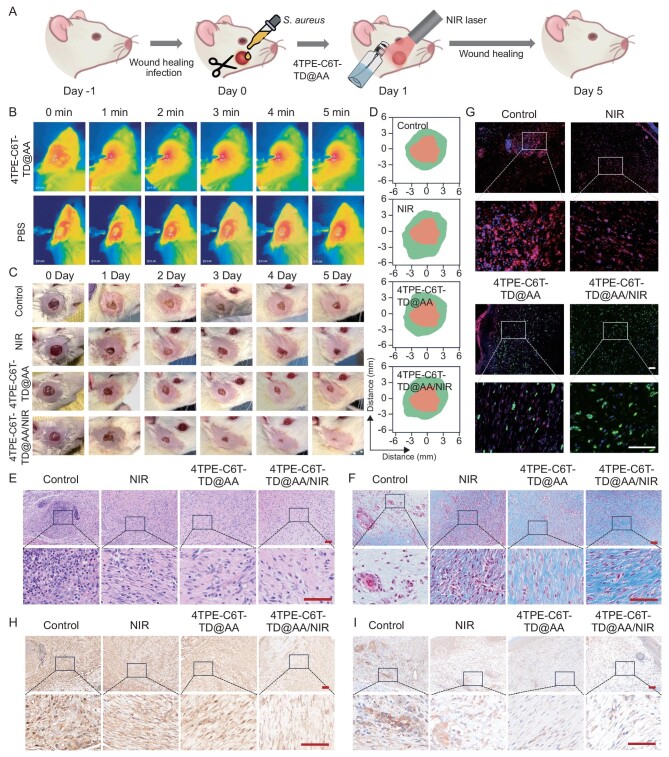
Synergistic PTT with 4TPE-C6T-TD@AA in a rat maxillofacial infection wound model. (A) Establishment of the rat maxillofacial infection wound model and its treatment schematic diagram. (B) Infrared thermal images of NIR and 4TPE-C6T-TD@AA/NIR groups for 5 min NIR laser irradiation, (C) photos of maxillofacial wounds during treatment and (D) visual images of preoperative and postoperative areas of wounds. (E) H&E staining images. Scale bar, 50 μm. (F) Masson staining images. Scale bar, 50 μm. (G) Immunofluorescence staining images, where CD86 shows red fluorescence, and CD206 shows green fluorescence. Scale bar, 50 μm. (H) Immunohistochemical staining images of IL-6 and (I) TNF-α. Scale bar, 50 μm.

After verifying the *in vitro* antibacterial and immunomodulatory functions, the healing effects of 4TPE-C6T-TD@AA were examined *in vivo* (Fig. 5A). Full-thickness wounds were created on the rats’ maxillofacial regions, infected with *S. aureus*, and subjected to various treatments (*n* = 3). The control group was treated with PBS, the 4TPE-C6T-TD@AA group was treated with pure NPs solution, and the NIR group was subjected to NIR laser irradiation. On the contrary, the experimental groups received 4TPE-C6T-TD@AA treatment followed by NIR laser irradiation. Infrared thermal images were captured during treatment (Fig. 5B), and wounds were photographed daily for 5 days postoperatively. While the tissue temperature increased by only 3.2°C following PBS treatment and NIR laser irradiation, the 4TPE-C6T-TD@AA/NIR group exhibited a remarkable increase of 14.5°C within 5 min of NIR illumination. This substantial temperature rise indicates effective stimulation of the PTT effect of 4TPE-C6T-TD@AA *in vivo* (Fig. S47). Photographs showed noticeable yellow pus oozing from the wound surface 24 hours after *S. aureus* infection. The wounds in the control group still presented significant purulent secretion on the 4th-day post-operation. Both weak NIR therapy and pure 4TPE-C6T-TD@AA with their anti-inflammatory effects, can alleviate the inflammatory reaction and promote healing to some extent. Instead, the wound tissue treated with the combined 4TPE-C6T-TD@AA/NIR therapy showed no obvious bacterial infection, with the wound nearly closed and healed (Fig. 5C and D). After 5 days of treatment, the 4TPE-C6T-TD@AA/NIR group showed a significant reduction in wound area to 7.8%, markedly smaller than that of other groups (Fig. S48). Bacterial cultures on agar plates confirmed the elimination of bacteria in the experimental group (Fig. S49). These results corresponded to the photothermal experiments *in vivo*, which showed that the high PCE of 4TPE-C6T-TD promotes efficient sterilization.

After 5 days of treatment, wound tissues were collected for histological analysis to assess the impact of 4TPE-C6T-TD@AA on wound healing. H&E staining (Fig. 5E) showed a substantial presence of neutrophils in the control group tissues. In contrast, both the NIR and 4TPE-C6T-TD@AA groups exhibited reduced inflammatory cell infiltration, with minimal inflammatory cells in the 4TPE-C6T-TD@AA/NIR group (Fig. S50). Moreover, newly formed collagen provided significant support for cell regeneration and wound healing. Masson staining results shown in Fig. 5F revealed fibrous connective tissue structure degradation due to inflammation, with the control group exhibiting the most severe damage to collagen fiber structure. As AA is a cofactor of collagen synthase, wound tissue treated with 4TPE-C6T-TD@AA showed increased collagen fiber content. When bacteria were eliminated and fibroblasts reduced the invasion from virulence factors, the 4TPE-C6T-TD@AA/NIR group displayed the densest collagen fiber density (Fig. S51). Result from the Gram staining was consistent with the observations from the bacterial spread plate assay. The control group harbored numerous bacteria, while deeply stained bacteria were scarcely observed in the 4TPE-C6T-TD@AA/NIR group (Fig. S52).

Following the impressive anti-inflammatory and antibacterial properties of 4TPE-C6T-TD@AA/NIR, we sought to delve deeper into the mechanisms involved. Macrophages are pivotal in the local immune response following tissue injury [48]. In wounds with persistent inflammation due to bacterial infection, the continuous aggregation of M1 macrophages creates a detrimental environment for tissue regeneration, while driving macrophage polarization to the M2 type can effectively regulate the inflammatory microenvironment of wounds [49,50]. Therefore, we performed immunofluorescence staining to evaluate the distribution of M1 and M2 macrophages, with CD86 identifying M1 macrophages and CD206 indicating M2 type. As shown in Fig. 5G, CD86 expression in the 4TPE-C6T-TD@AA/NIR group decreased compared to the control group. Conversely, the percentage of CD206 was substantially higher in the experimental group compared to the other groups (Fig. S53). These findings highlight the role of 4TPE-C6T-TD@AA/NIR in bacterial eradication and modulating the immune microenvironment to facilitate the wound repair process. Immunohistochemical staining further revealed the expression of immune-related factors within wound tissue. Sections containing pro-inflammatory biomarkers IL-6 and TNF-α in the control group showed considerable brown cells, indicative of an inflammatory state. In contrast, both the NIR and 4TPE-C6T-TD@AA groups exhibited reduced brown cell counts, with only a few detected in the 4TPE-C6T-TD@AA/NIR group (Fig. 5H and I; Fig. S54). Conversely, the proportion of positively stained cells for anti-inflammatory biomarkers IL-10 and TGF-β was negatively correlated with the degree of inflammation in the sections, with the 4TPE-C6T-TD@AA/NIR group showing the highest expression of brown cells (Figs S55–S57). To further investigate the mechanisms of wound repair facilitated by 4TPE-C6T-TD@AA/NIR treatment, we performed immunohistochemical staining for α-SMA and CD31 (Figs S58 and S59). CD31-positive staining revealed an increase in angiogenesis, suggesting improved vascularization in the treated wounds. Additionally, the 4TPE-C6T-TD@AA/NIR-treated group exhibited significantly more α-SMA positive cells, indicating enhanced myofibroblast activity and wound contraction compared to the control group. These findings support the notion that 4TPE-C6T-TD@AA/NIR not only modulates the immune microenvironment but also promotes tissue repair through enhanced vascularization and wound contraction, contributing to accelerated healing.

## CONCLUSION

In summary, we designed and synthesized two NIR PTAs by leveraging the strong electron-withdrawing capacity of BBTD and incorporating four TPA or TPE moieties at their periphery to facilitate nonradiative decay. With its superior light-capturing ability, 4TPE-C6T-TD demonstrated high photothermal efficiency under 808 nm laser irradiation, making it the preferred candidate for PTT. After encapsulating 4TPE-C6T-TD in liposomes, AA was incorporated on the liposome surface to regulate oxidative stress and sustain the inflammatory response in infected wounds. *In vitro* experiments revealed that 4TPE-C6T-TD@AA disrupts the normal physiological metabolic functions of bacteria and targets their self-defense and repair mechanisms under 808 nm laser irradiation, and >90% of *S. aureus* and *E. coli* were eliminated within 5 min. Additionally, 4TPE-C6T-TD@AA can remove biofilms by inhibiting bacterial adhesion and killing bacteria deep inside the biofilm. Thus, it effectively inhibits biofilm formation and removes mature biofilms through NIR synergy. Furthermore, the antioxidant properties of 4TPE-C6T-TD@AA efficiently eliminate excess ROS wound tissue cells. When applied to maxillofacial infection wounds in rats, 4TPE-C6T-TD@AA exhibits potent antibacterial and anti-inflammatory effects *in vivo*. Upon NIR exposure, it rapidly eradicated bacteria and modulated the immune microenvironment of the wound tissue. This dual-action approach not only accelerates collagen synthesis and effectively controls inflammation but also significantly enhances the healing and repair of the wound tissue. In conclusion, 4TPE-C6T-TD@AA presents a promising solution for treating maxillofacial infectious wounds.

## Supplementary Material

nwae426_Supplemental_File
