# Supplementary material for: A tactfully designed photothermal agent collaborating with ascorbic acid for boosting maxillofacial wound healing
Source: Natl Sci Rev. 2024 Nov 26;12(2):nwae426. doi: 10.1093/nsr/nwae426 (PMC11737384; doi:10.1093/nsr/nwae426)
Supplement: nwae426_Supplemental_File [file nwae426_supplemental_file.pdf]

Supporting Information

# A Tactfully Designed Photothermal Agent Collaborating with Ascorbic Acid for Boosting Maxillofacial Wound Healing

Yuxin Qian,[1,#] Yiting Gao,[3,#] Dong Wang,[2,#] Shixuan Zhang,[1] Qiuxia Luo,[2] Guogang Shan,[3] Mengmeng Lu,[1,*] Dingyuan Yan,[2,*] Ben Zhong Tang[2,4,*] and Ming Zhang,[1,*]

[1]The Affiliated Stomatological Hospital of Nanjing Medical University. State Key Laboratory Cultivation Base of Research, Prevention and Treatment for Oral Diseases. Jiangsu Province Engineering Research Center of Stomatological Translational Medicine, Nanjing Medical University, Nanjing 210029, China;

[2]Center for AIE Research, Guangdong Provincial Key Laboratory of New Energy Materials Service Safety, College of Materials Science and Engineering, Shenzhen University, Shenzhen 518060, China;

[3]Institute of Functional Material Chemistry and National & Local United Engineering Lab for Power Battery, Faculty of Chemistry, Northeast Normal University, Changchun 130024, China;

[4]School of Science and Engineering, Shenzhen Institute of Aggregate Science and Technology, The Chinese University of Hong Kong, Shenzhen (CUHK-Shenzhen), Guangdong 518172, China

[*]**Corresponding Authors.** E-mails: lumm@njmu.edu.cn; yandingyuan@szu.edu.cn; tangbenz@ust.hk; mzhan@njmu.edu.cn

[#]Equally contributed to this work.

# Experimental section

## Main materials

Commercially available chemicals, such as 1-(4-broMophenyl)-1,2,2-triphenylethylene, 1-(4-aminophenyl)-1,2,2-triphenylethene, 4-bromotriphenylamine, 4-aminotriphenylamine, 2-bromo-4-hexylthiophene, P(*t*-Bu)$_3$ HBF$_4$, *t*BuONa, Pd$_2$(dba)$_3$, *n*-BuLi, ClSnBu$_3$, and P(*o*-tol)$_3$, *et al.* were obtained from Adamas, Energy, TCI and Bide and used as received unless otherwise stated.

## Instruments for experiments

Reactions were monitored with analytical thin-layer chromatography (TLC) on silica. $^1$H NMR and $^{13}$C NMR data were recorded on Bruker nuclear resonance (400 MHz, 500 MHz, and 600 MHz) spectrometers using CDCl3 as the deuterated solvent unless otherwise specified, respectively. Chemical shifts ($\delta$) are given in ppm relative to TMS. UV–Vis–NIR absorption spectra were measured on a PerkinElmer Lambda 950 spectrophotometer. PL spectra were recorded on the FluoroMax$^+$ fluorescence spectrophotometer of HORIBA.

## General procedure for the synthesis of D4TPE-C6T-TD and D4TPA-C6T-TD

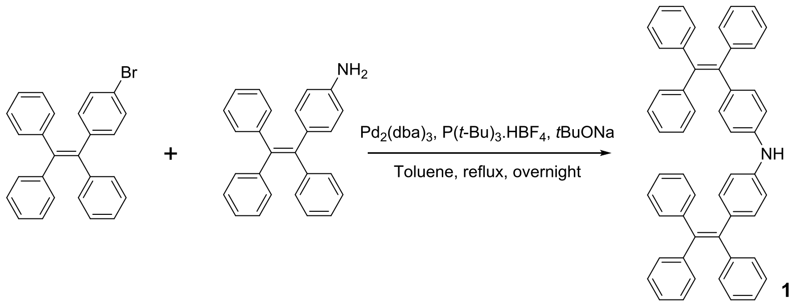


1

**Synthetic route of 1**: Compound 1-(4-bromophenyl)-1,2,2-triphenylethylene (0.41 g, 1 mmol), 1-(4-aminophenyl)-1,2,2-triphenylethene (0.35 g, 1 mmol), Pd$_2$(dba)$_3$ (0.046 g, 0.05 mmol), P(*t*-Bu)$_3$ HBF$_4$ (0.029 g, 0.1 mmol), and *t*BuONa (0.097 g, 1 mmol) were placed in a reaction flask, and then 30 mL of toluene solution was added under nitrogen atmosphere. The mixture reacts at 115 ℃ for 24 h. After the reaction is completed and cooled to room temperature, the solvent is removed by vacuum rotary evaporation. The crude product is redissolved with dichloromethane and then washed three times with water. After drying the organic layer with anhydrous sodium sulfate and removing the solvent under reduced pressure, the yellow compound **1** (570 mg, yield of 85%) was purified through a silica gel column using petroleum ether/dichloromethane (v: v=5:1) as eluent. $^1$H NMR (500 MHz, Chloroform-*d*) δ 7.32 (d, 1H), 7.07-6.95 (m, 30H), 6.82-6.73 (m, 8H). $^{13}$C NMR (101Hz, Chloroform-*d*) δ 145.30, 145.16, 145.10, 142.94, 141.97, 140.53, 136.13, 133.05, 132.30, 132.22, 132.18, 128.42, 128.30, 127.04, 126.91, 126.86, 116.83.

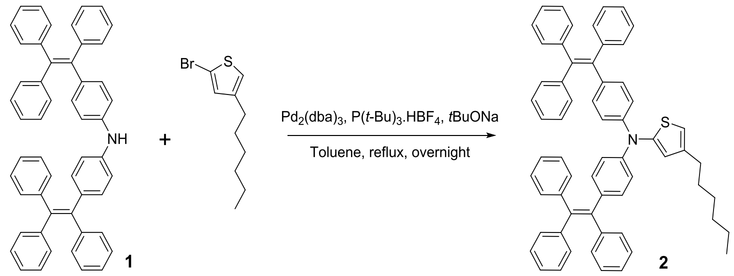


**Synthetic route of 2**: Place compound **1** (0.677 g, 1 mmol), 2-bromo-4-hexylthiophene (0.247 g, 1 mmol), $Pd_2(dba)_3$ (0.046 g, 0.05 mmol), $P(t\text{-}Bu)_3 \cdot HBF_4$ (0.029 g, 0.1 mmol), and *t*BuONa (0.097 g, 1 mmol) into a reaction flask, and then add 30 mL of toluene solution under nitrogen atmosphere. The mixture reacts at 115 ℃ for 24 h. After the reaction is completed and cooled to room temperature, the solvent is removed by vacuum rotary evaporation. The crude product is redissolved with dichloromethane and then washed three times with water. After drying the organic layer with anhydrous sodium sulfate and removing the solvent under reduced pressure, the yellow compound **2** (720 mg, yield of 86%) was purified through a silica gel column using petroleum ether/dichloromethane (v: v=10:1) as eluent. $^1$H NMR (500 MHz, Chloroform-*d*) δ 7.17-7.08 (m, 30H), 6.93-6.92 (m, 4H), 6.87-6.86 (m, 4H), 6.59 (d, 1H), 6.53 (d, 1H), 2.55-2.53 (t, 2H), 1.65-1.60 (m, 2H), 1.40-1.35 (m, 6H), 0.97-0.94 (t, 2H). $^{13}$C NMR (101Hz, Chloroform-*d*) δ 150.42, 145.99, 144.07, 143.95, 143.76 141.78, 140.71, 140.57, 138.04, 132.07, 131.52, 131.48, 131.46, 127.72, 126.51, 126.46, 126.40, 122.77, 121.32, 114.91, 31.77, 31.11, 30.21, 29.09, 22.72, 14.24.

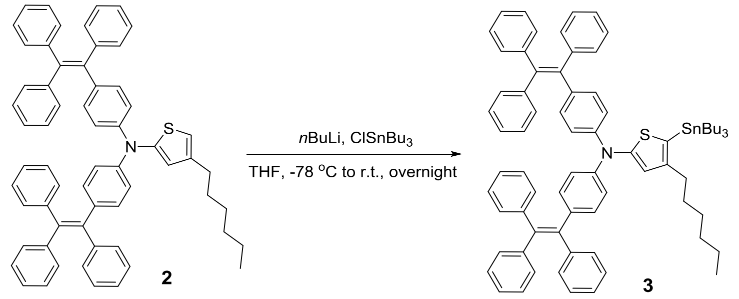


**Synthetic route of 3**: Dissolve compound **2** (0.844 g, 1 mmol) in 20 mL of dry THF, cool to -78 ℃ under nitrogen atmosphere, and then add 1.2 M *n*-BuLi (0.5 mL, 1.2 mmol) dropwise. Stir the mixture at -78 ℃ for 1 h, then add $ClSnBu_3$ (0.36 mL, 1.2 mmol) and slowly heat the reaction to room temperature for another 12 h. After the reaction is completed, quench the reaction with a saturated potassium fluoride solution, pour the mixture into water, and then extract twice with ethyl acetate. The organic layer is dried with anhydrous sodium sulfate and concentrated through a rotary evaporator. The obtained yellow oily compound **3** does not require further purification and can be directly subjected to the next reaction.

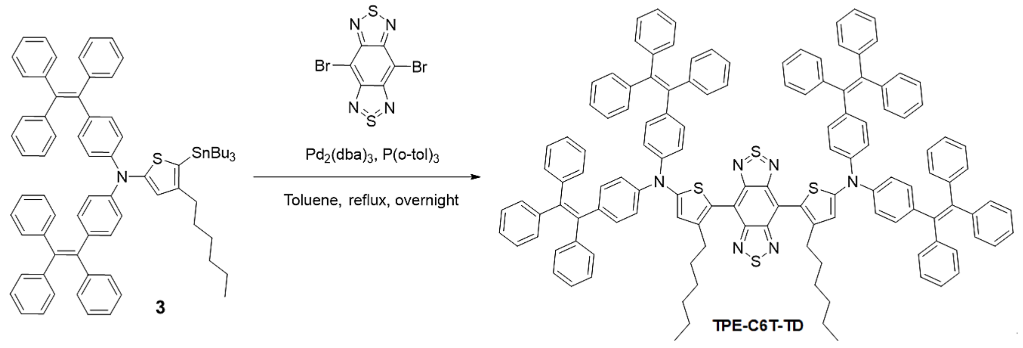


**Synthetic route of 4TPE-C6T-TD**: Under a nitrogen atmosphere, mix all obtained compounds **3**, 4,7-dibromobenzo [1,2-c-4,5-c'] bis([1,2,5]thiadiazole) (BBTD) (0.106 g, 0.3 mmol), $Pd_2(dba)_3$ (0.029 g, 0.03 mmol), P(*o*-tol)$_3$ (0.071 g, 0.3 mmol), and anhydrous toluene (4 mL) at 120 ℃ for 24 h. After the reaction is completed and cooled to room temperature, the solvent is removed by vacuum rotary evaporation. The crude product is redissolved with dichloromethane and then washed three times with water. After drying the organic layer with anhydrous sodium sulfate and removing the solvent under reduced pressure, the dark green compound 4TPE-C6T-TD (57 mg, yield of 10%) was purified through a neutral alumina column using petroleum ether/dichloromethane (v: v=5:1) as the eluent. $^{1}$H NMR (500 MHz, THF-$d_8$) δ 7.10-7.02 (m, 52H), 7.00-6.98 (m, 8H), 6.96-6.95 (m, 4H), 6.93-6.91 (m, 4H), 6.68 (d, 2H), 2.51-2.49 (d, 4H), 1.56-1.50 (m, 4H), 1.13-1.07 (m, 12H), 0.75-0.72 (d, 4H). $^{13}$C NMR (101Hz, THF-$d_8$) δ 154.53, 154.11, 146.68, 144.92, 144.73, 144.52, 144.09, 141.85, 141.53, 139.84, 133.03, 132.18, 132.16, 128.47, 128.44, 128.38, 127.23, 127.13, 124.57, 122.95, 122.27, 116.28, 32.50, 31.57, 30.99, 30.01, 23.41, 14.36. **HRMS (ESI)** calculated for $C_{130}H_{104}N_6S_4$ $[M+H]^+$: 1877.7233; Found: 1877.7189.

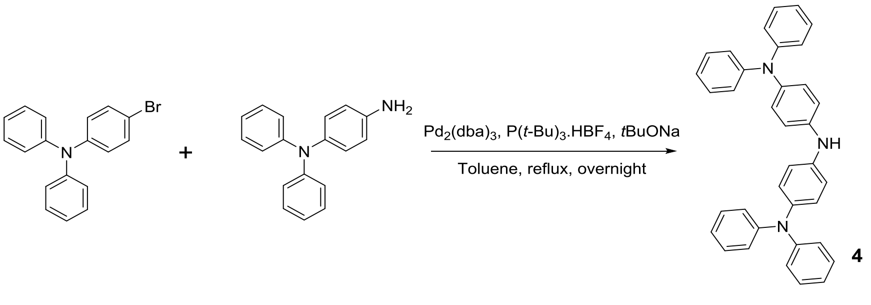


**Synthetic route of 4:** Compounds 4-bromotrianiline (0.32 g, 1 mmol), 4-aminotriphenylamine (0.25 g, 1 mmol), $Pd_2(dba)_3$ (0.046 g, 0.05 mmol), P(*t*-Bu)$_3$ HBF$_4$ (0.029 g, 0.1 mmol), and *t*BuONa (0.097 g, 1 mmol) were placed in a reaction flask, and then 30 mL of toluene solution was added under nitrogen atmosphere. The mixture reacts at 115 ℃ for 24 h. After the reaction is completed and cooled to room temperature, the solvent is removed by vacuum rotary evaporation. The crude product is redissolved with dichloromethane and washed three times with water. After drying the organic layer with anhydrous sodium sulfate and removing the solvent under reduced

pressure, the yellow compound **4** (452 mg, yield of 90%) was purified through a silica gel column using petroleum ether/dichloromethane (v: v=5:1) as eluent. $^{1}$H NMR (500 MHz, *THF-$d_8$*) δ 7.29 (d, 1H), 7.17-7.15 (m, 8H), 7.02-7.00 (m, 12H), 6.97-6.96 (m, 4H), 6.89-6.87 (m, 4H). $^{13}$C NMR (101Hz, *THF-$d_8$*) δ 149.24, 141.41, 141.06, 129.76, 127.75, 123.67, 122.48, 118.99.

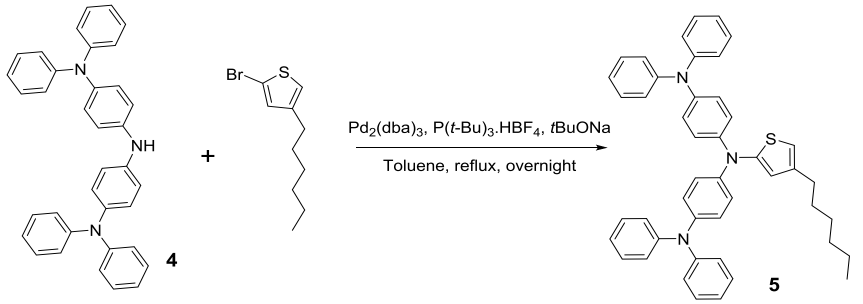


**Synthetic route of 5:** Compound **4** (0.503 g, 1 mmol), 2-bromo-4-hexylthiophene (0.247 g, 1 mmol), $Pd_2(dba)_3$ (0.046 g, 0.05 mmol), P(*t*-Bu)$_3$ HBF$_4$ (0.029 g, 0.1 mmol), and *t*BuONa (0.097 g, 1 mmol) were placed in a reaction flask, and then 30 mL of toluene solution was added under nitrogen atmosphere. The mixture reacts at 115 ℃ for 24 h. After the reaction is completed and cooled to room temperature, the solvent is removed by vacuum rotary evaporation. The crude product is redissolved with dichloromethane and then washed three times with water. After drying the organic layer with anhydrous sodium sulfate, the solvent was removed under reduced pressure. The yellow solid **5** (590 mg, yield 88%) was purified by silica gel column using petroleum ether/dichloromethane (v: v=10:1) as the eluent. $^{1}$H NMR (500 MHz, Chloroform-*d*) δ 7.17-7.13 (m, 8H), 7.01-6.96 (m, 8H), 6.94-6.91 (m, 4H), 6.90-6.88 (m, 8H), 6.51-6.46 (d, 2H), 2.45-2.42 (t, 2H), 1.52-1.49 (m, 2H), 1.25-1.20 (m, 6H), 0.81-0.78 (t, 3H). $^{13}$C NMR (101Hz, Chloroform-*d*) δ 150.91, 147.90, 143.27, 142.59, 141.92, 129.19, 125.36, 123.71, 123.12, 122.38, 122.02, 114.48, 31.69, 31.08, 30.13, 29.01, 22.64, 14.13.

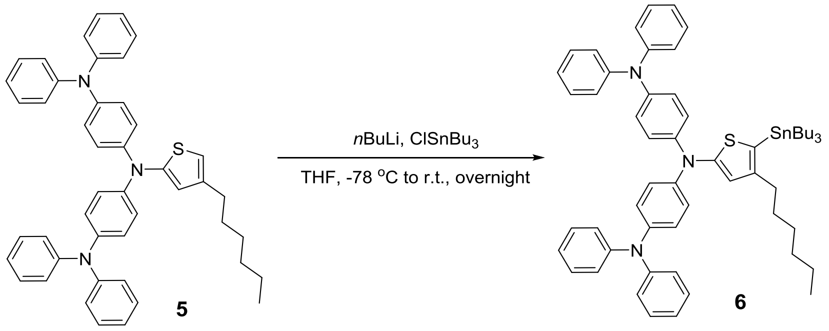


**Synthetic route of 6**: Dissolve compound **5** (0.67 g, 1 mmol) in 20 mL of dry THF, cool to -78 ℃ under nitrogen atmosphere, and add 1.2 M *n*-BuLi (0.5 mL, 1.2 mmol) dropwise. Stir the mixture at -78 ℃ for 1 h, then add $ClSnBu_3$ (0.36 mL, 1.2 mmol) and slowly heat the reaction to room temperature for another 12 h. After the reaction is completed, quench the reaction with a saturated potassium fluoride solution, pour the mixture into water, and then extract twice with

ethyl acetate. The organic layer is dried with anhydrous sodium sulfate and concentrated through a rotary evaporator. The obtained yellow oily compound **6** does not require further purification and can be directly subjected to the next reaction.

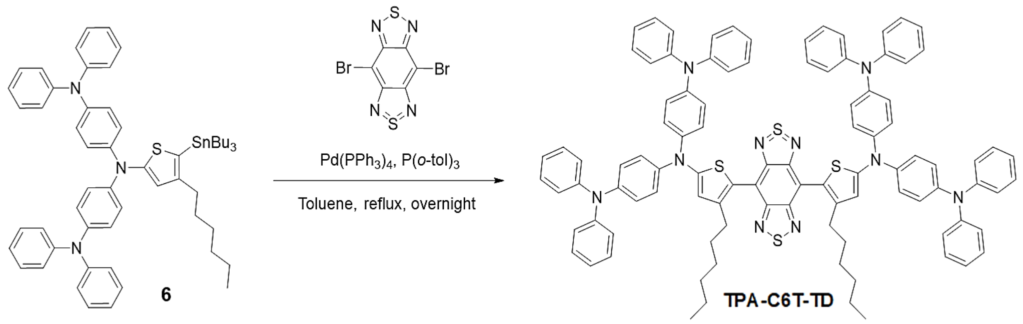


**Synthetic route of 4TPA-C6T-TD:** Under a nitrogen atmosphere, all obtained mixtures of compound **6**, BBTD (0.106 g, 0.3 mmol), $Pd_2(dba)_3$ (0.029 g, 0.03 mmol), P(*o*-tol)$_3$ (0.071 g, 0.3 mmol), and anhydrous toluene (4 mL) were stirred at 120 ℃ for 24 h. After the reaction is completed and cooled to room temperature, the solvent is removed by vacuum rotary evaporation. The crude product is redissolved with dichloromethane and then washed three times with water. Dry the organic layer with anhydrous sodium sulfate and remove the solvent under reduced pressure. Purify the rust-red solid 4TPA-C6T-TD (45 mg, yield 10%) using petroleum ether/dichloromethane (v: v=5:1) as the eluent through a neutral alumina column. $^1$H NMR (500 MHz, Chloroform-*d*) δ 7.15-7.12 (m, 8H), 7.01-7.00 (m, 8H), 6.91-6.89 (m, 4H), 6.80-6.76 (m, 8H), 6.51-6.47 (d, 2H), 2.45-2.42 (t, 2H), 1.52-1.49 (m, 2H), 1.25-1.20 (m, 6H), 0.81-0.78 (t, 3H). $^{13}$C NMR (101Hz, THF-$d_8$) δ 153.17, 147.88, 143.62, 142.65, 129.04, 128.96, 128.78, 124.78, 124.19, 123.83, 123.51, 122.70, 122.66, 122.46, 31.52, 30.08, 29.66, 29.05, 22.42, 13.37. HRMS (ESI) calculated for $C_{98}H_{84}N_{10}S_4$ $[M+H]^+$: 1529.5836; Found: 1529.58318.

**Preparation of 4TPE-C6T-TD@AA liposomes**

1,2-distearoyl-*sn*-glycerol-3-phosphate ethanolamine PEG acid (DSPE-PEG$_{2000}$-COOH, 1mg) was stirred in $H_2SO_4$ (98%) until completely dissolved in a three-necked bottle, then added AA (1 mg), stirring until completely dissolved (25 ℃, 48 h). Poured the reaction product into crushed ice, precipitated crystals, and extracted with solvent solution, and washed it with purified water until neutral. Distilled under reduced pressure to obtain a crude product, and recrystallized with chloroform to obtain DSPE-PEG$_{2000}$@AA. Mixed 4TPE-C6T-TD (1 mg) and DSPE-PEG$_{2000}$@AA (1 mg) in DMSO, then drop them dropwise into 9 mL of deionized water under ultrasonic condition and continue ultrasonic for 7 min to synthesize 4TPE-C6T-TD@AA.

**Photothermal performance measurement**

The aqueous solution of 4TPE-C6T-TD@AA with different concentrations (0, 25, 50, 75, and 100 μg/mL) was continuously exposed to 808 nm laser at 0.8 W/cm$^2$ and 4TPE-C6T-TD@AA with 100 μg/mL was continuously exposed to 808 nm laser at different power density (0.2, 0.4, 0.6, 0.8 and 1 W/cm$^2$). The temperature was measured every 30 s for 5 min. The corresponding infrared thermal images of different samples were also recorded. In the 'On and Off Experiment', ICG and 4TPE-C6T-TD@AA (100 μg/mL) were selected as experimental objects, and the laser was turned off 5 minutes after the laser was turned on for 5 minutes, and this process was repeated for 5 times. Real-time temperature and infrared thermal images were recorded during the experiment. The photothermal conversion efficiency in an aqueous solution was calculated according to the cooling curve.

**Reactive oxygen species (ROS) scavenging assay**

First, the antioxidant ability of the 4TPE-C6T-TD@AA was examined by 1,1-diphenyl-2-trinitrophenylhydrazine (DPPH) radical scavenging assay. Briefly, DPPH ethanol solution (0.1 mmol/L) was prepared. Afterward, samples were submerged in the DPPH solution and kept at 37 ℃ in a dark environment for 40 min. The absorbance of each sample at 517 nm was measured every 5 min using a spectrophotometer. After that, the absorbance spectra of each sample were recorded. The formula used to calculate the scavenging rate was:

$$\text{Scavenging Rate} = \left(1 - \frac{\mathrm{A_t}}{\mathrm{A_i}}\right) \times 100\% \qquad (1)$$

where $A_i$ signifies the initial absorbance of the DPPH solution, and $A_t$ signifies the absorbance at 517 nm of DPPH after a 30-minute interaction with the sample. Subsequently, the capacity of the materials to neutralize ROS was evaluated using the total antioxidant capacity kit [2,2'-azino-bis(3-ethylbenzthiazoline-6-sulfonic acid, ABTS]. The test samples were submerged in the ABTS solution. Then the experimental process is similar to the DPPH radical scavenging assay, but the absorbance at 414 nm is used as $A_t$ to calculate the scavenging rate. Then, $H_2O_2$ is mixed with $FeSO_4$ to make it undergo a Fenton reaction and generate OH. When salicylic acid is added to the reaction system, OH can be captured, and 3- hydroxy salicylic acid and 5- hydroxy salicylic acid can be generated. After adding each sample into the reaction system, the absorbance spectra were recorded by spectrophotometer. Electron paramagnetic resonance (EPR) was used to detect the ability of 4TPE-C6T-TD@AA to scavenge superoxide anion ($O^{2-}$) free radicals. The total reaction system is 30 μl, which contains 0.08 M DMPO, 5 mM EDTA, 0.3 M riboflavin, 2 mM DETAPAC, and 5 μl PBS buffer or 100 μg/mL 4TPE-C6T-TD@AA. After

rapid mixing, it is sucked into a Shi Ying capillary and put into a resonance cavity. After 90 s, ESR signals are measured and spectrograms are recorded.

**Bacteria culture**

The strains used in the bacteria test in this study are Gram-positive S. aureus (ATCC 49295) and Gram-negative E. coli (ATCC 25922). LB solid medium (Jiangsu Keygen Biotech Corp., Ltd, Nanjing) is used for bacterial solid culture. According to the product instructions, 40 g dry powder is dissolved in 1 l deionized water, and sterilized at 120 ℃ for 30 min at high temperature and high pressure. Immediately after sterilization, the solid medium is poured into a bacterial Petri dish and stored at 4 ℃ after cooling and solidification. After the bacteria were inoculated or coated on a flat plate, they were placed in a 37 ℃ microbial incubator for solid culture. LB liquid medium was used for bacterial liquid culture. According to the product instructions, 25 g dry powder was dissolved in 1 l deionized water, sterilized at 120 ℃ for 30 min, and stored at 4 C. After bacteria were inoculated into the liquid culture medium, they were incubated for 8~12 h in a 37 ℃ shaker at 200 rpm, and the logarithmic growth period could be reached. After two generations of culture, the follow-up experiments were carried out.

**Colony Forming Unit (CFU) Assay**

The colony-forming unit (CFU) assay was employed to enumerate viable bacteria in solution [1]. Briefly, a series of tenfold gradient dilutions of bacteria suspension was made. 10 μL of each diluted solution was then dropped on the LB agar plate (n = 3). After 24 h, the bacteria colony count on plates was recorded. ∑N is the sum of colony counts on all replicate plates for the same dilution. D is the dilution factor for the dilution used. The total number of live bacteria per mL can be calculated by following below formula:

$$\text{CFU per mL} = \frac{\sum N}{0.01D} \quad (2)$$

Then the bacterial suspensions of different treatment groups were diluted $10^{-5}$~$10^{-7}$, and coated on flat plates. The plates were incubated for 12 h at 37 ℃, and the experiments were done in triplicate. The CFUs were recorded, and the percent inhibition was calculated based on the following formula:

$$\text{Bacterial viability} = \frac{\text{CFU}_{\text{treated}}}{\text{CFU}_{\text{untreated}}} \times 100\% \quad (3)$$

**LIVE/DEAD bacterial viability assay**

The staining was done using the Invitrogen™ Live/Dead BacLight™ Bacterial Viability Kit. The Live/Dead Viability is a two-dye system consisting of Syto9 green fluorescent dye and propidium iodide (PI) red fluorescent dye. Both nucleic acid dyes can be used to differentiate live from dead bacteria. PI penetrates damaged bacterial membranes while Syto9 stains bacteria with intact cell membranes. Thus, live cells will be stained in green, and dead cells in red. According to the manufacturer's recommendation, equal parts of the Syto9 and PI were combined (Thermo Fisher Scientific, Waltham, MA, USA). Bacteria in different treatment groups were collected and incubated with the dye mixture at room temperature in the dark for 15 min. The bacteria were then observed using a fluorescence microscope.

**Morphology changes of bacteria**

The morphology change of bacteria with different treatments was explored using SEM. S. aureus and E. coli ($10^6$ CFU/mL) were cultured with 100 μL $H_2O$ or 4TPE-C6T-TD@AA (100 μg/mL). 808 nm NIR laser irradiation (1.0 W/cm$^2$) was then applied to the mixed solution for 10 min. After irradiation, 2.5% glutaraldehyde solution was added for fixing at 4 ℃ overnight. The samples were then washed with deionized water three times and dehydrated in an alcohol series (20%, 40%, 60%, 80%, 100%, 5 min each). Subsequently, bacteria with different treatments were collected, centrifuged, redispersed, and dried for TEM observation.

**Anti-biofilm test**

To investigate the in vitro anti-biofilm ability of 4TPE-C6T-TD@AA, 200 μL S. aureus and E. coli ($10^6$ CFU/mL) were inoculated in a 96-well plate and incubated at 37 ℃ to form biofilms. After 24 h, the culture medium was washed with $H_2O$ three times to rinse the planktonic bacteria before adding 100 μL $H_2O$ or 4TPE-C6T-TD@AA (100 μg/mL). The solution was then applied with 808 nm NIR laser irradiation (1.0 W/cm$^2$) or not for 5 min. After different treatments, biofilms were stained with PI for 5 min and SYTO 9 for 15 min and observed with a laser scanning confocal microscopy.

Quantitative Real-Time PCR Analysis

Total RNA was extracted from bacteria with different treatments using the RNAiso Plus reagent (Takara, USA). The RNA concentration was quantified with a NanoDrop 2000 spectrophotometer (Thermo Scientific, USA). The reverse transcription reaction and qRT-PCR were performed with the PrimeScript RT reagent Kit (Takara, USA) and SYBR Master mix

(Takara, USA). Primer sequences are listed in Table S1 (Supporting Information). GAPDH was applied to normalize the gene expression level.

**Cell culture**

NCTC clone 929 (L929) cells, RAW 264.7, and Human Oral Keratinocytes (HOK) cells were purchased from the Type Culture Collection of the Chinese Academy of Sciences (Shanghai, China). These cells were cultured in Dulbecco's modified Eagle medium (DMEM) complete medium under identical culture conditions.

**In vitro cytotoxicity study**

CCK-8 assay was adopted to evaluate the in vitro cytotoxicity of 4TPE-C6T-TD@AA. Briefly, L929 and HOK cells were seeded onto 96-well plates of a density of 6000 cells per well and incubated overnight. Then fresh cell culture media containing a series of concentrations (0, 25, 50, 75, and 100, 200μg/mL) of 4TPE-C6T-TD@AA were added and incubated with the cells for 24 h, respectively. The medium was then replaced with fresh medium containing CCK-8 (10%). After another 1 h incubation, the viability of cells was analyzed by measuring the absorption value at 450 nm using a microplate BioRad reader. Data were presented as mean ± standard deviation (SD).

**In vivo cytotoxicity study**

4TOE-C6T-TD or PBS was injected into the tail vein of mice (n = 3). 24 hours after intravenous injection, mice were sacrificed to harvest the main organs, and then the organs were fixed in 4% paraformaldehyde for 24 hours at 4 ℃, and H&E staining was performed to detect pathological changes in the heart, liver, spleen, lung, and kidney.

**Detection of Intracellular ROS**

ROS levels in the cells were further detected using DCFH-DA (Molecular Probes, USA). The adherent cells were stained with immunofluorescence. Briefly, RAW 264.7 and L929 cells after treatment were washed with pre-warmed PBS and stained by 50 μM DCFH-DA for 30 min. Then the fluorescent images were taken with a fluorescence microscope. Then the ROS levels of RAW 264.7 and L929 cells were measured by flow cytometry. Each group of cells was harvested by centrifugation at 1000 rpm for 5 min after washing with PBS twice. Then, cells were incubated with50 μM DCFH-DA for 30 min at 37 ℃ in the dark. The cells were then analyzed using flow cytometry (n = 3).

**Assessment of macrophage polarization**

The mean fluorescence intensity of the cell surface molecules was assessed by FCM. The cells in each group were collected in 1.5 mL cold PBS and centrifuged at 1500 rpm for 5 min, twice. Subsequently, the suspension was discarded and the pellet was resuspended with PBS. The Fc receptor sites were blocked with anti-mouse CD16/CD32 purified antibodies (BioLegend, San Diego, USA) on ice for 20 min to prohibit non-specific binding before the cells were incubated with the specific fluorescent-labeled antibody. Afterward, the membrane surface molecules were stained with 3 µL/test of FITC-conjugated anti-mouse CD86 MAb (eBiosience, San Diego, USA) or APC-conjugated anti-mouse CD206 MAb (BioLegend) for 30 min at room temperature in the dark as per the manufacturer's instructions. The samples were washed thrice with PBS and suspended in 4% paraformaldehyde. Use BD FACSAria™ Fusion for detection, then the FCM analysis was applied with the FlowJo software. The experimental steps of qRT-PCR are the same as above. Primer sequences are listed in Table S2 (Supporting Information), and 16s was applied to normalize the gene expression level.

**PTT of 4TPE-C6T-TD@AA in a rat maxillofacial infection wound model**

All animal experiments were performed according to the "Principles of Laboratory Animal Care" (NIH publication No. 86-23) and the guidelines for the Animal Care and Use Committee, Nanjing Medical University (IACUC-2303044). At day 0, fifteen female SD rats (150–170 g) were anesthetized by isoflurane. Then the maxillofacial region of rats was made a full-thickness skin wound with a diameter of 8 nm using scissors and inoculated with *S. aureus* suspension ($10^6$ CFU/mL). After 24 h post-infection, the rat maxillofacial infection wound model was successfully built. The infected rats were randomly divided into four groups (three mice per group) and received different treatments including $H_2O$ and 4TPE-TD@AA (100 µg/mL), then applied with 808 nm NIR laser irradiation (1.0 W/cm$^2$) or not for 5 min. Infrared thermal images were acquired by using infrared imaging devices, and the appearance of the wound was recorded by mobile phone from day 1 to 5.

**Histological study**

The collected wound tissue was kept in formalin, embedded in paraffin, sectioned, and stained with hematoxylin-eosin (H&E), Masson, gram, immunofluorescence, and immunohistochemical staining for pathology analysis.

**Statistical analysis**

Student's t-test and ANOVA were used for comparison between two groups and among multiple

groups, respectively. Statistical analyses were conducted using Microsoft Excel and Origin 2021 software. A value of $p < 0.05$ was considered statistically significant.

## Supporting figures

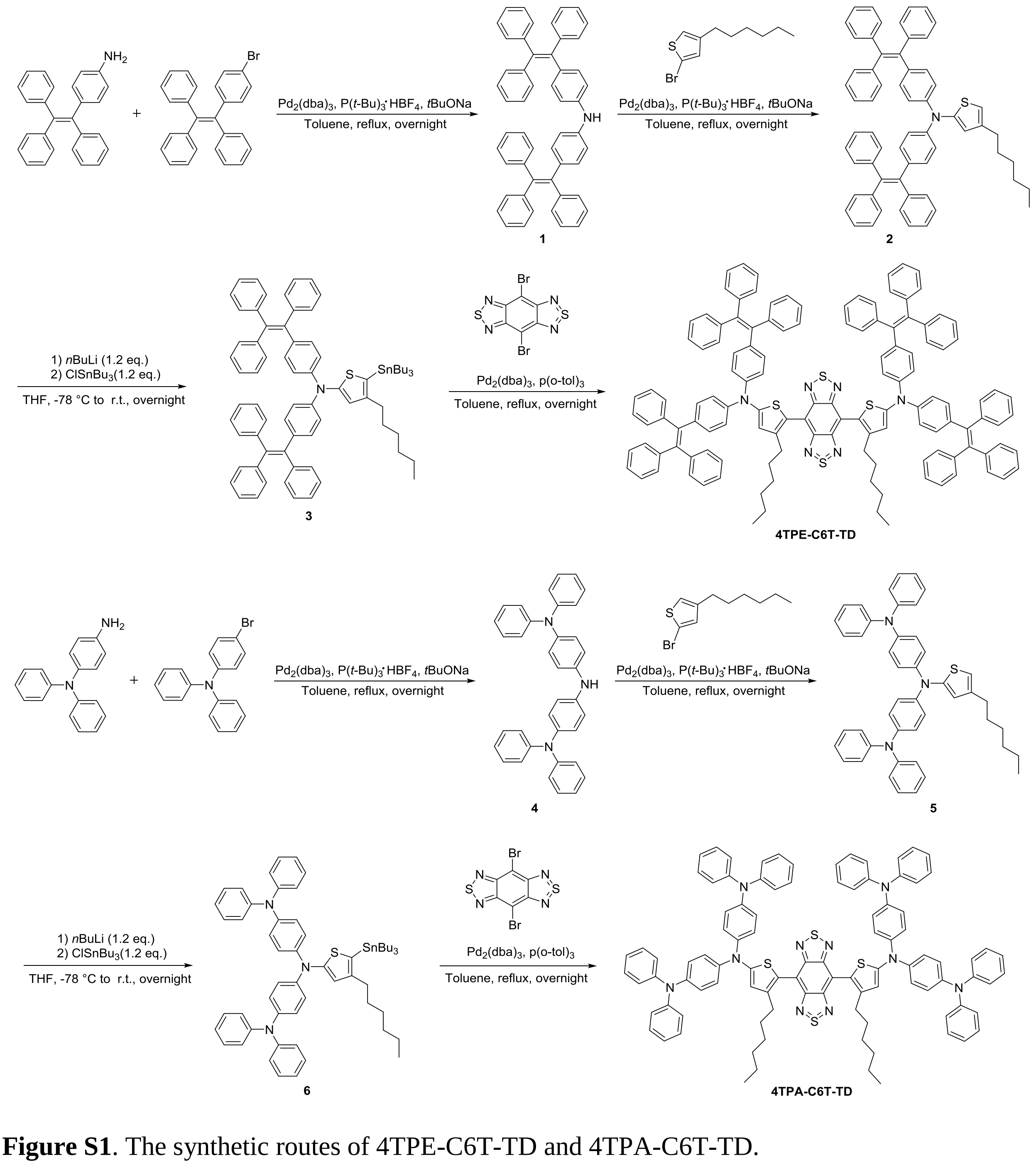


**Figure S1**. The synthetic routes of 4TPE-C6T-TD and 4TPA-C6T-TD.

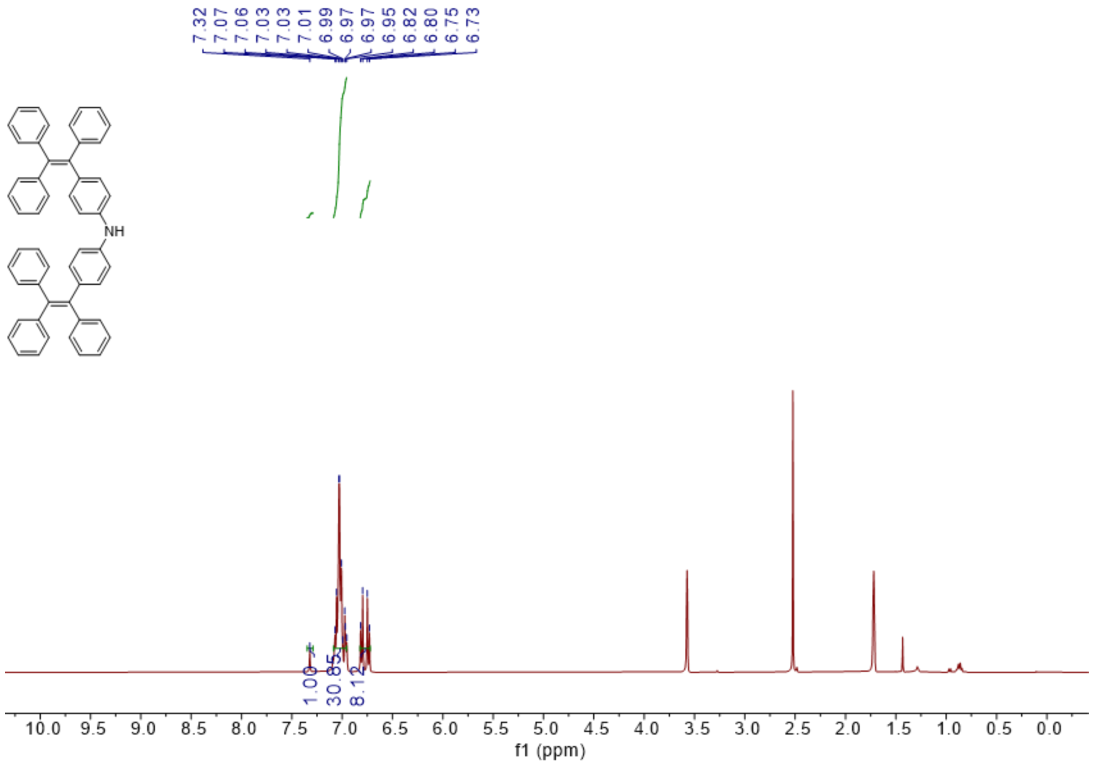

**Figure S2.** $^{1}$H NMR spectrum of **1**.

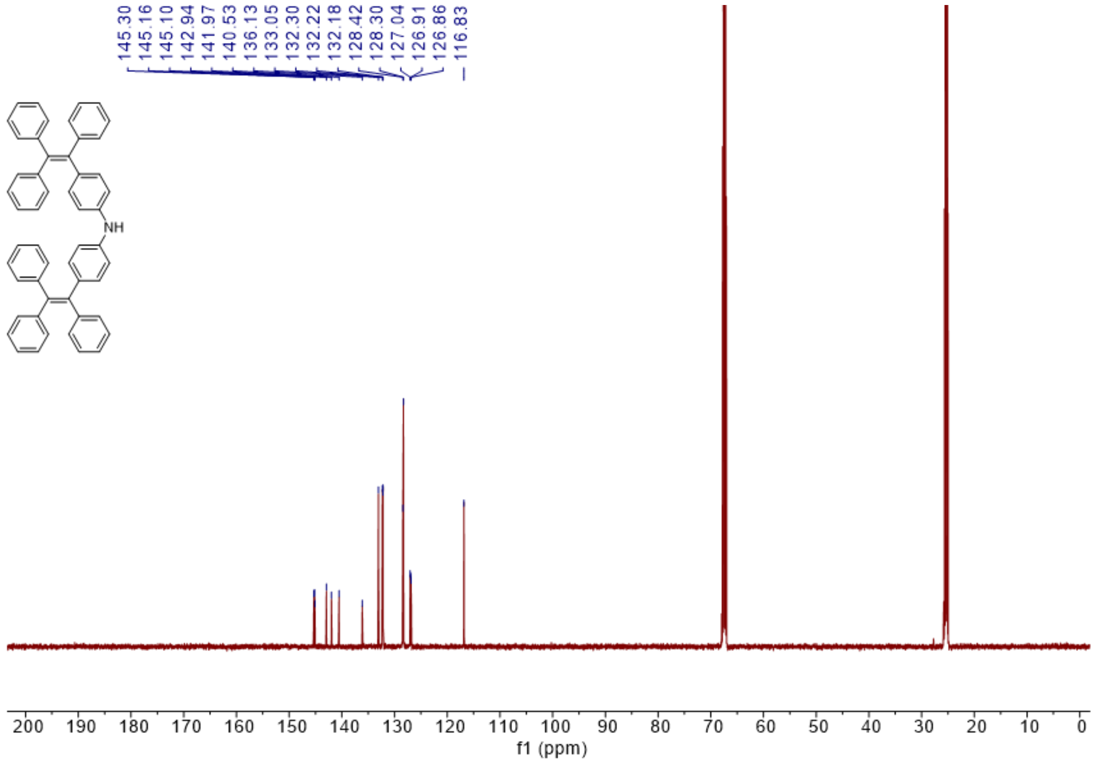

**Figure S3.** $^{13}$C NMR spectrum of **1**.

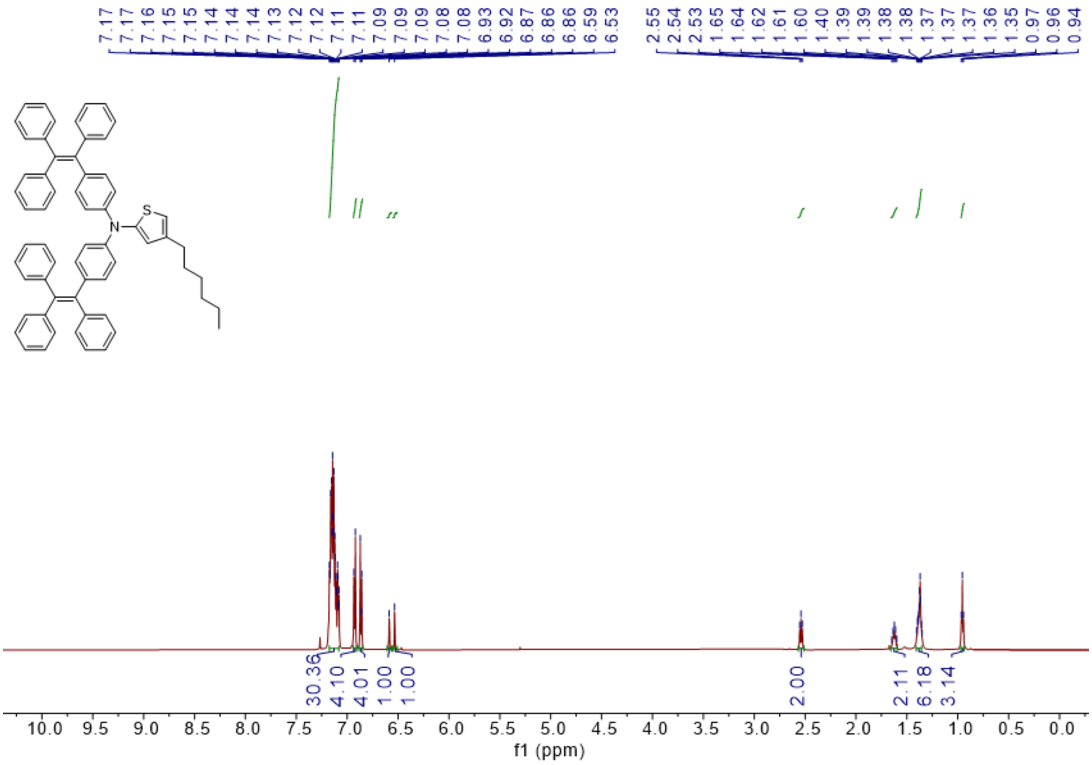

**Figure S4.** $^{1}H$ NMR spectrum of **2**.

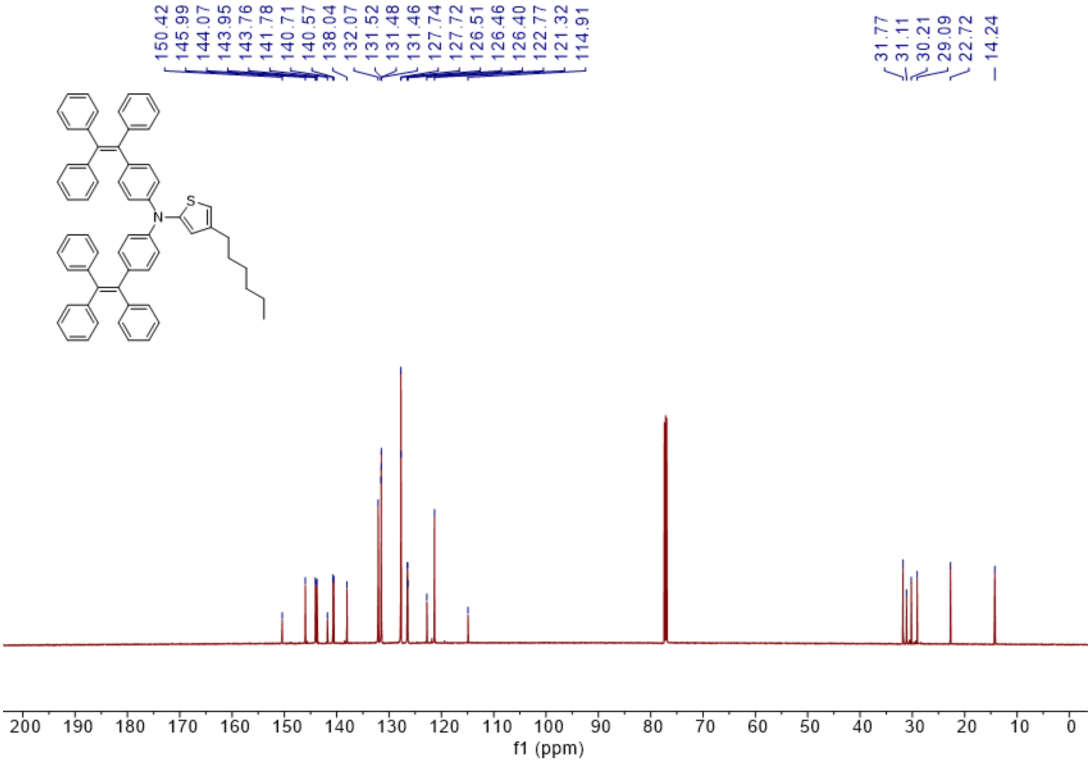

**Figure S5.** $^{13}C$ NMR spectrum of **2**.

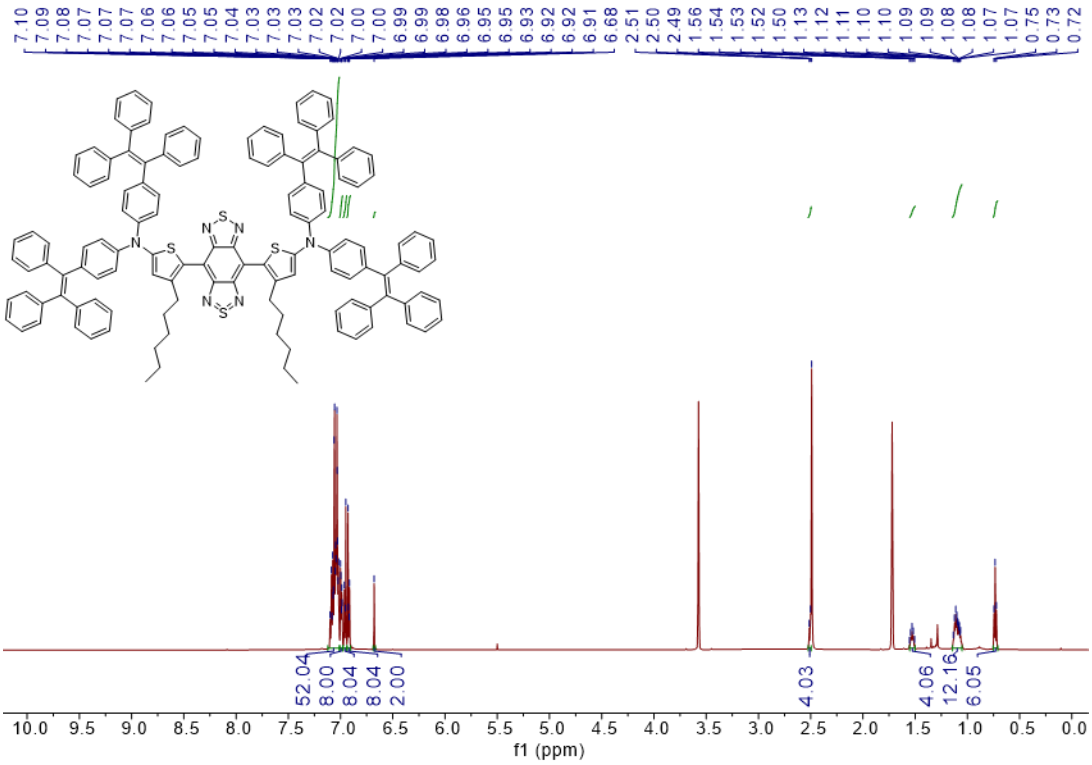


**Figure S6.** $^{1}$H NMR spectrum of 4TPE-C6T-TD.

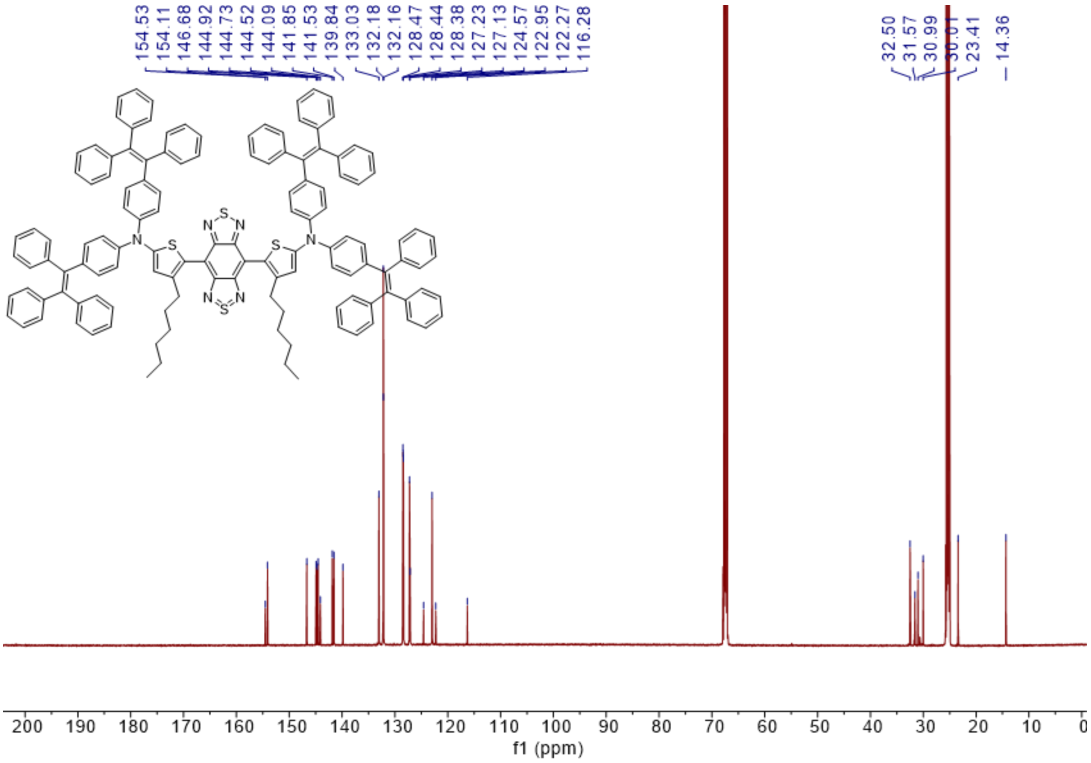


**Figure S7.** $^{13}$C NMR spectrum of 4TPE-C6T-TD.

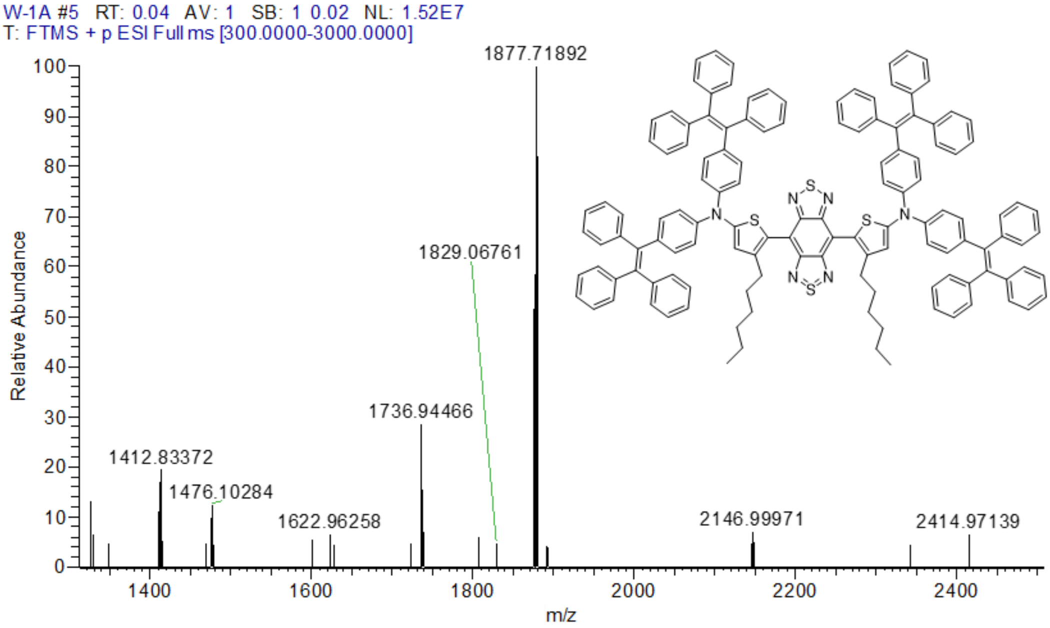


**Figure S8.** HRMS spectrum of compound 4TPE-C6T-TD.

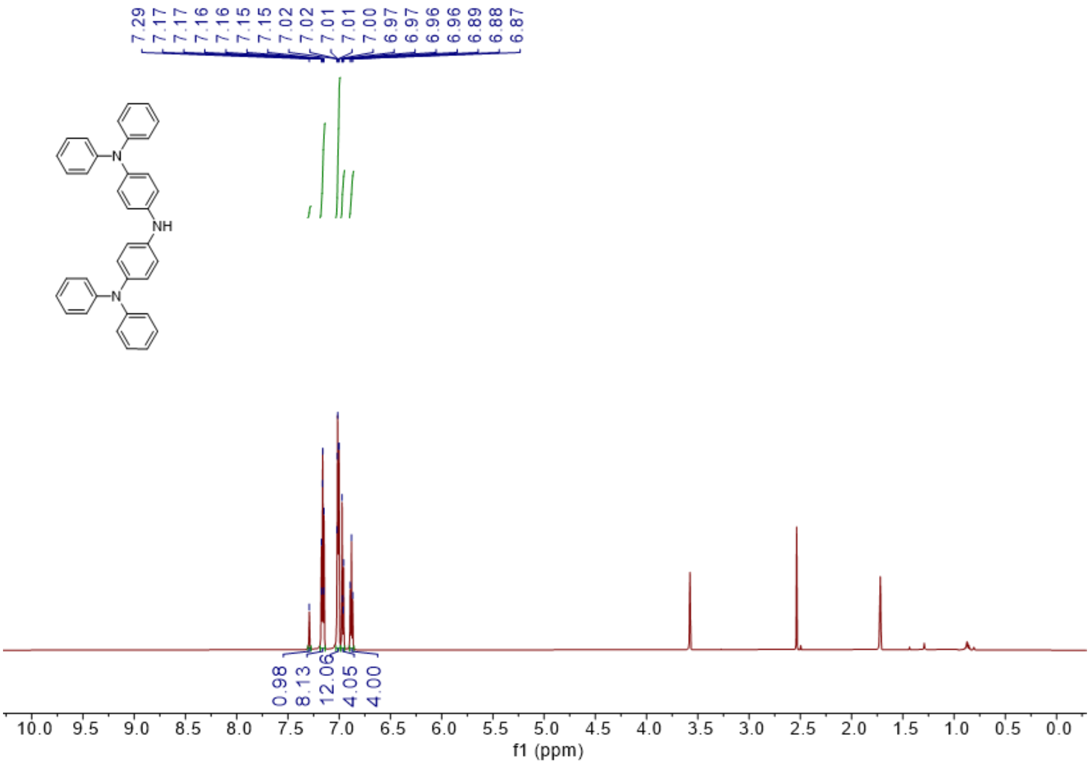


**Figure S9.** $^{1}$H NMR spectrum of **4**.

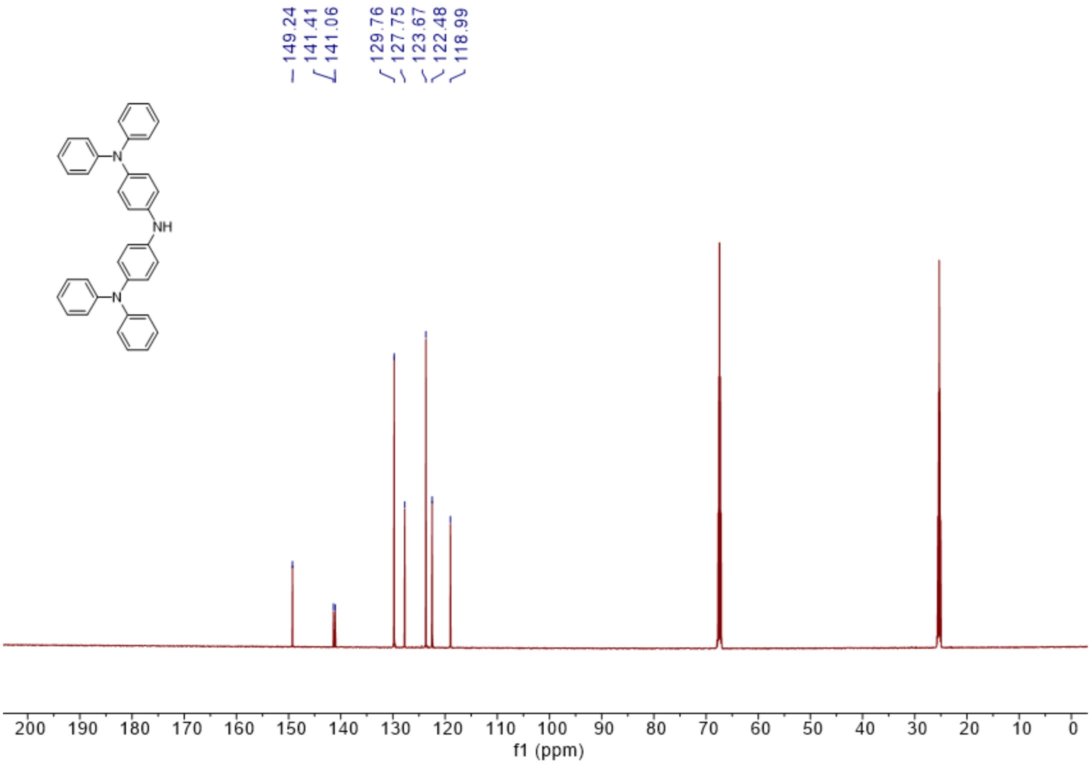


**Figure S10.** $^{13}$C NMR spectrum of **4**.

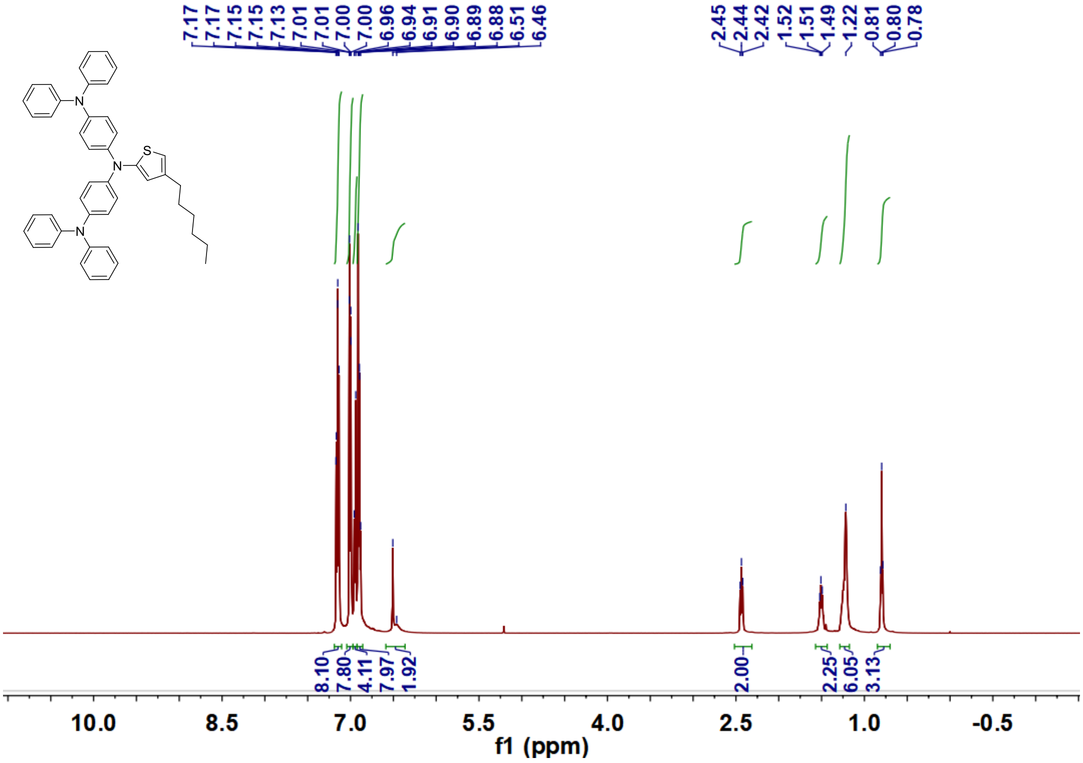


**Figure S11.** $^{1}$H NMR spectrum of **5**.

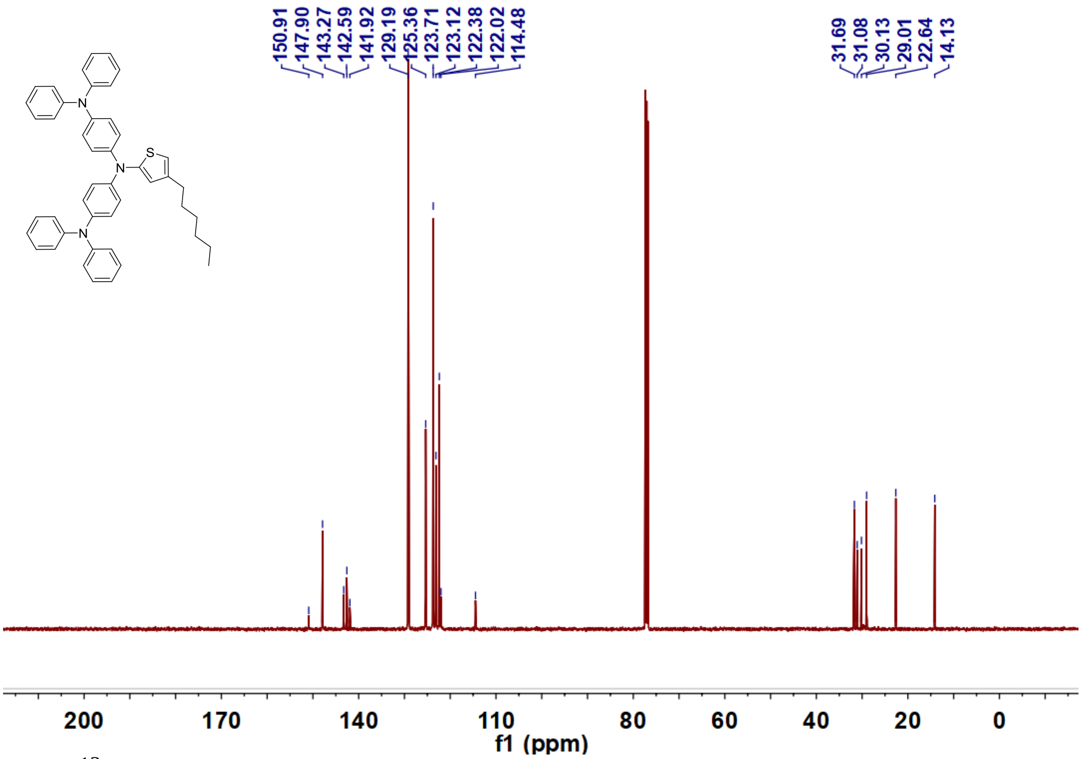


**Figure S12.** $^{13}$C NMR spectrum of **5**.

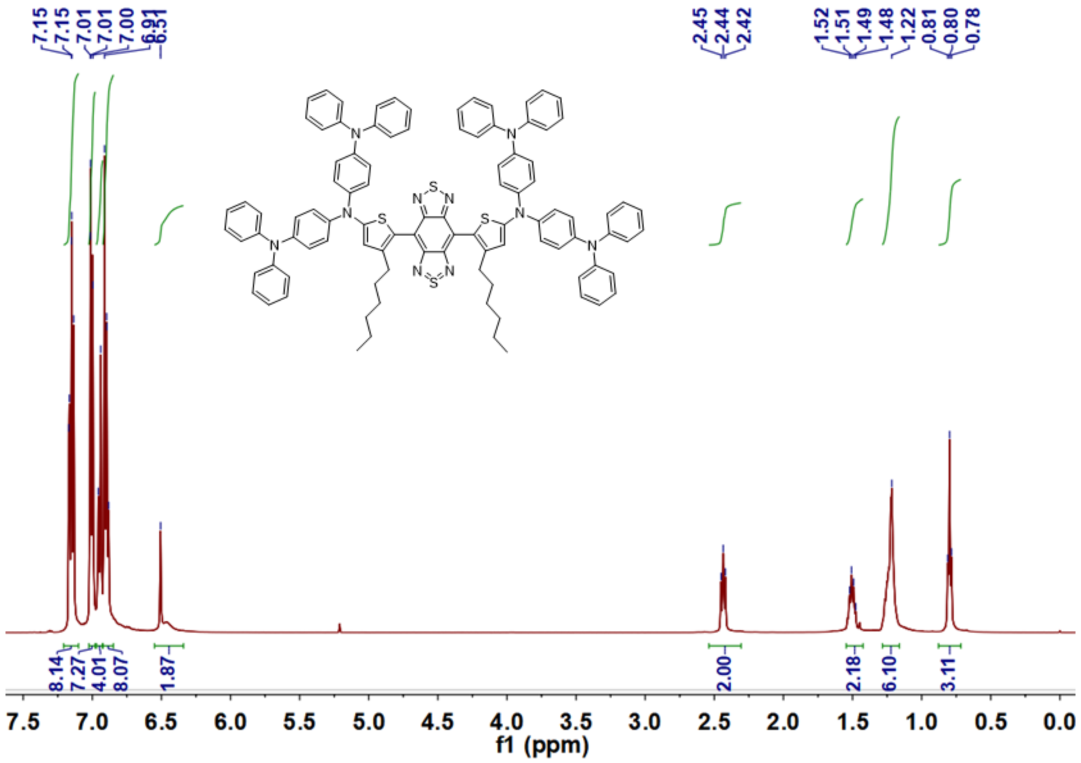


**Figure S13.** $^{1}$H NMR spectrum of 4TPA-C6T-TD.

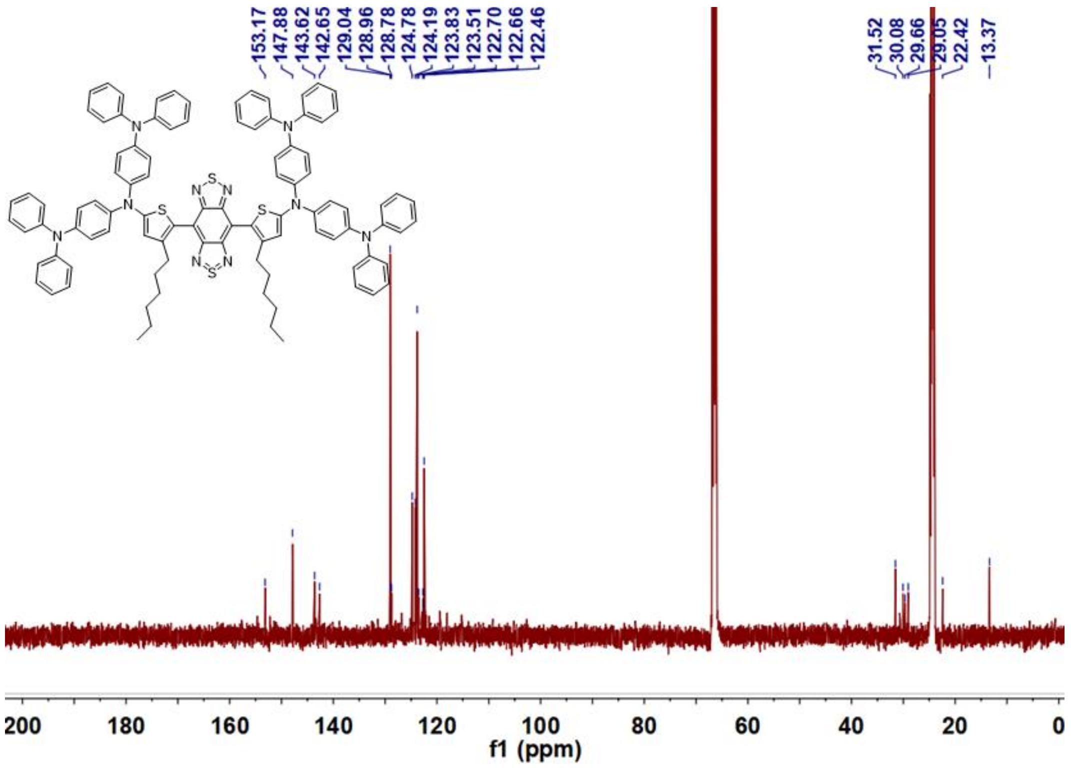


**Figure S14.** $^{13}C$ NMR spectrum of 4TPA-C6T-TD.

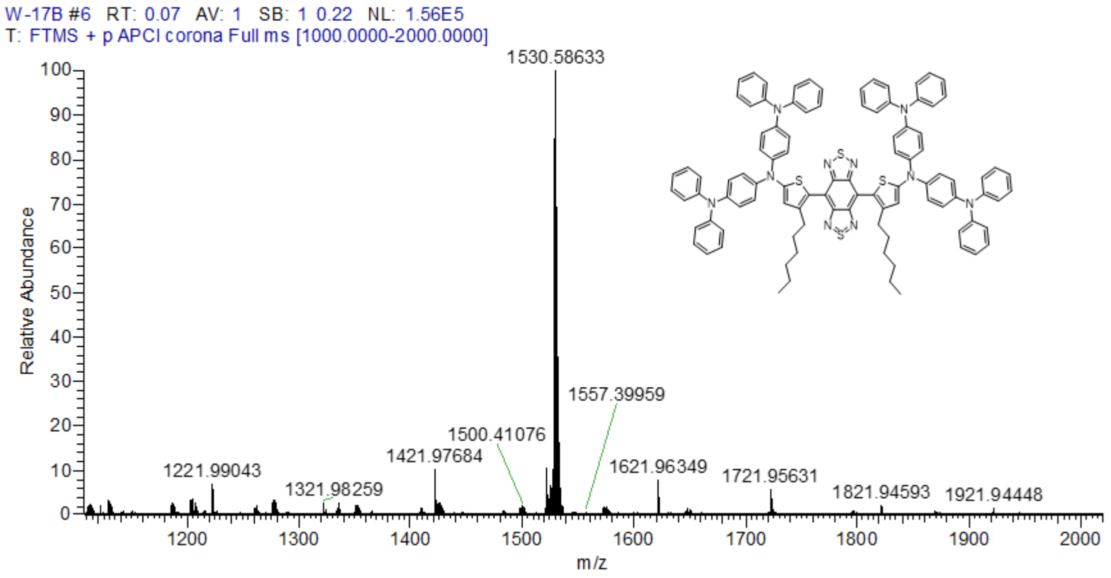


**Figure S15.** HRMS spectrum of compound 4TPA-C6T-TD.

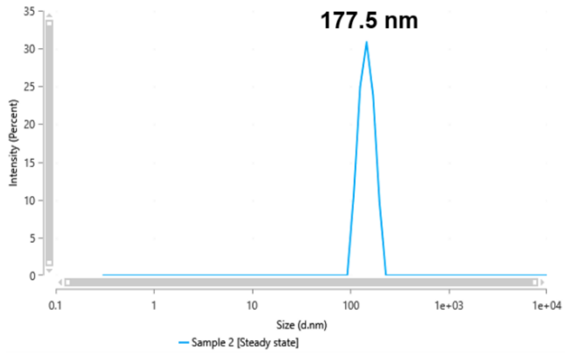


**Figure S16.** The particle size distribution of 4TPE-C6T-TD@AA was characterized using Dynamic Light Scattering (DLS).

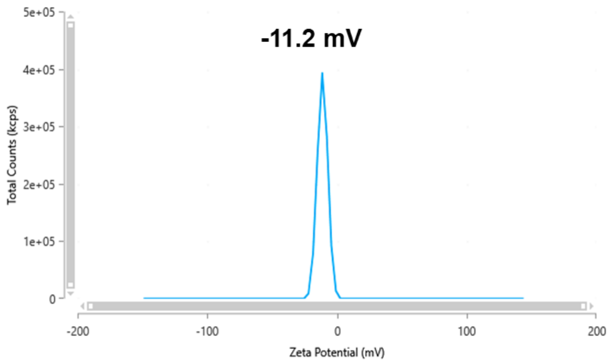


**Figure S17.** Zeta potential analysis of 4TPE-C6T-TD@AA.

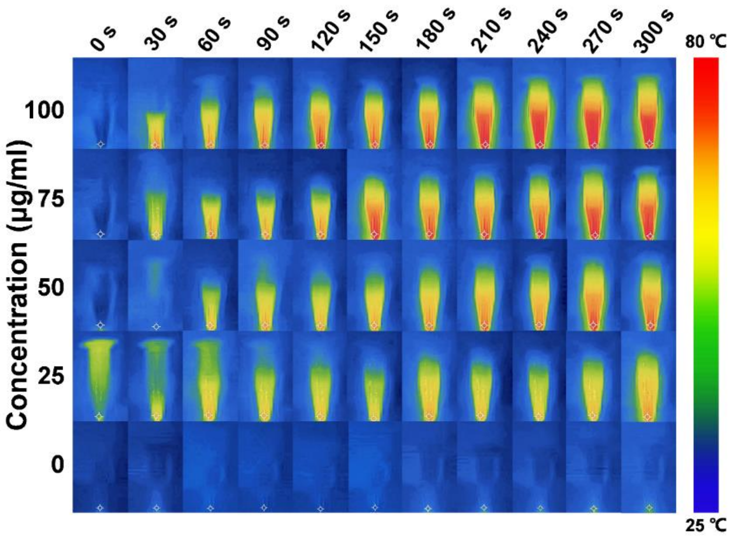


**Figure S18.** *In vitro* infrared thermal images of 4TPE-C6T-TD@AA with different concentrations (0, 25, 50, 75, and 100 μg/mL) under 808 nm laser irradiation (0.8 W/cm$^2$).

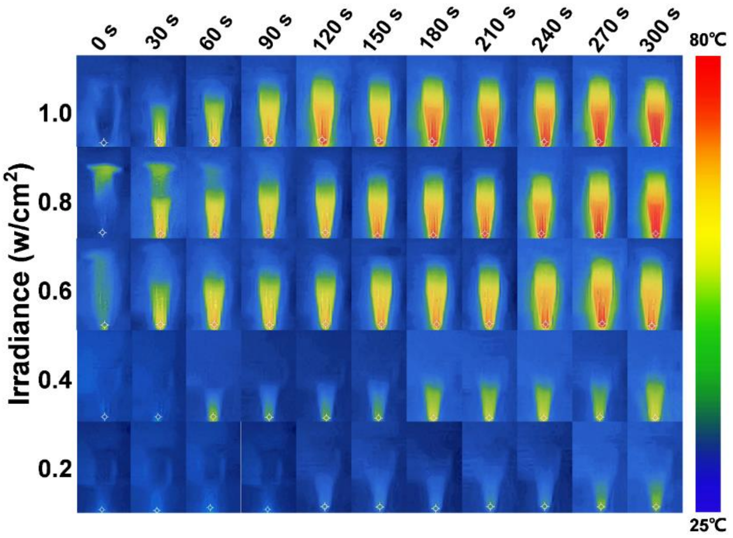


**Figure S19.** *In vitro* infrared thermal images of 4TPE-C6T-TD@AA (100 μg/mL) with different power densities (0.2, 0.4, 0.6, 0.8 and 1.0 W/cm$^2$).

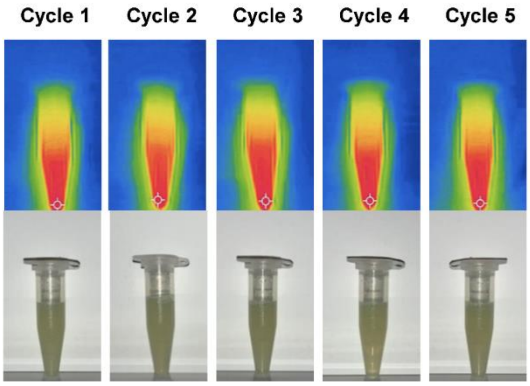


**Figure S20.** Infrared thermal images and photographs of photothermal conversion cycling test of 4TPE-C6T-TD@AA.

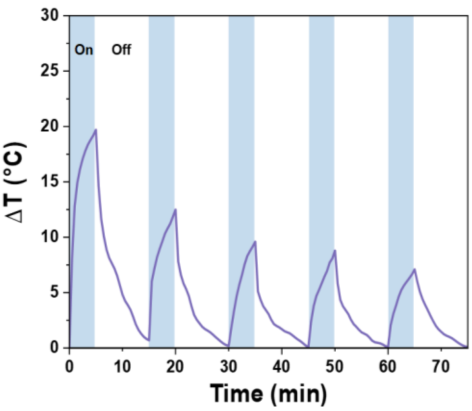


**Figure S21.** Heating and cooling curves of photothermal conversion cycling test of ICG.

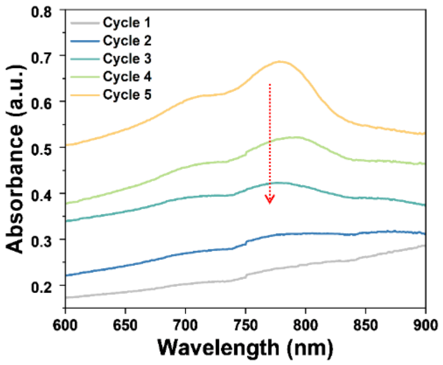


**Figure S22.** The absorption spectra of photothermal conversion cycling test of ICG.

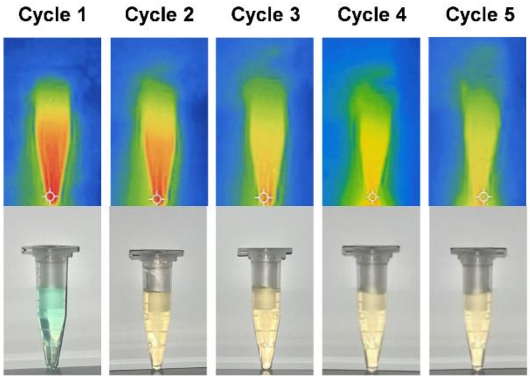


**Figure S23.** Infrared thermal images and photographs of photothermal conversion cycling test of ICG.

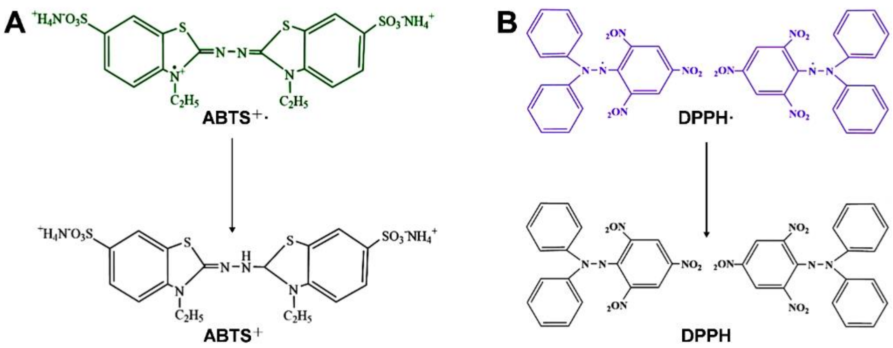


**Figure S24.** Schematic diagram of (A) ABTS and (B) DPPH experiment principle.

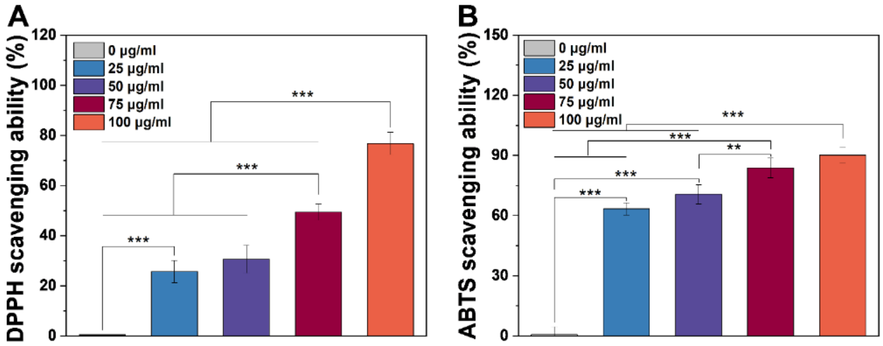


**Figure S25.** Scavenging efficiency of (A) DPPH test and (B) ABTS test evaluating the total ROS scavenging capacity of 4TPE-C6T-TD@AA ($n$ = 3). Statistical analysis was performed using one-way ANOVA with Tukey's post-test. **$p < 0.01$ and ***$p < 0.001$.

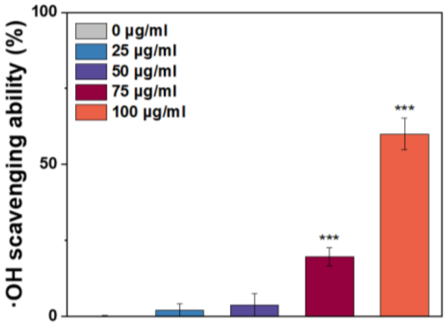


**Figure S26.** The efficiency of removing $^{\cdot}$OH by 4TPE-C6T-TD@AA at different concentrations ($n$ = 3). Statistical analysis was performed using one-way ANOVA with Tukey's post-test. ***$p < 0.001$.

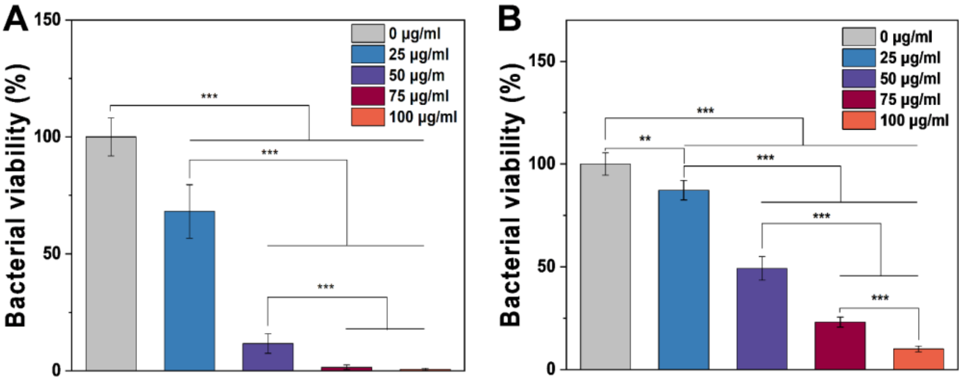


**Figure S27.** Bacterial viability of (A) *S. aureus* and (B) *E. coli* after treatment with different concentrations of 4TPE-C6T-TD@AA under 808 nm laser irradiation ($n$ = 3). Statistical analysis was performed using one-way ANOVA with Tukey's post-test. **$p < 0.01$ and ***$p < 0.001$.

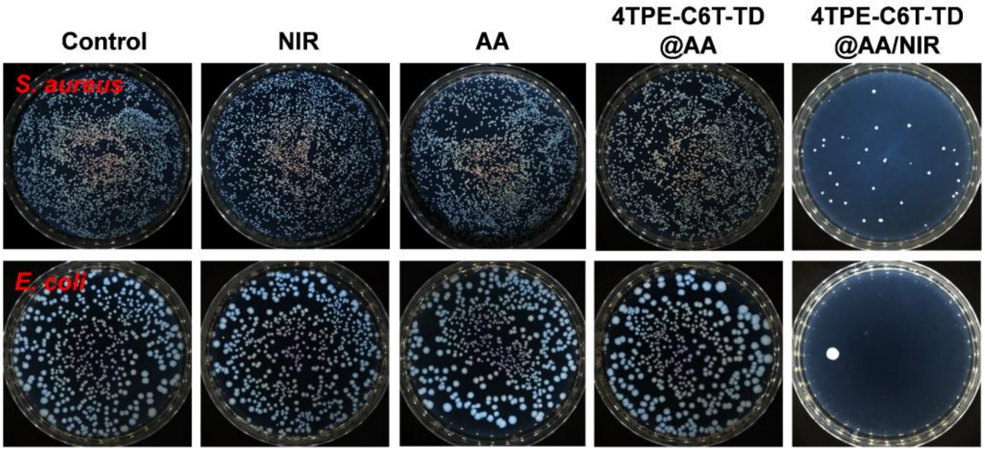


**Figure S28.** Typical photographs of bacterial colonies on the agar plates with different treatment.

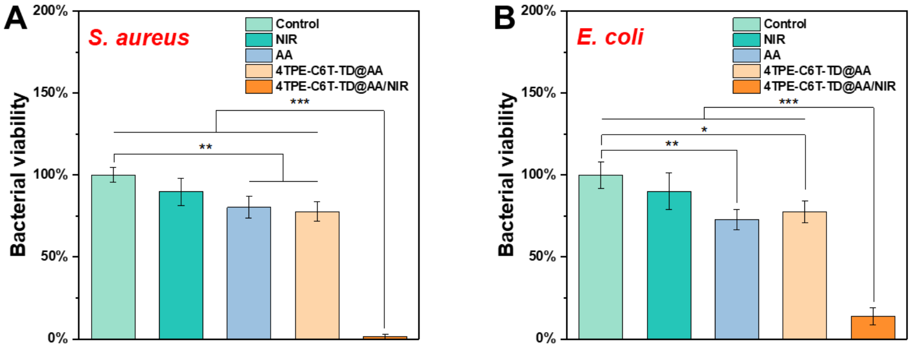


**Figure S29.** Antibacterial rates against (A) *S. aureus* and (B) *E. coli* ($n$ = 3). Statistical analysis was performed using one-way ANOVA with Tukey's post-test. *$p < 0.05$, **$p < 0.01$ and ***$p < 0.001$.

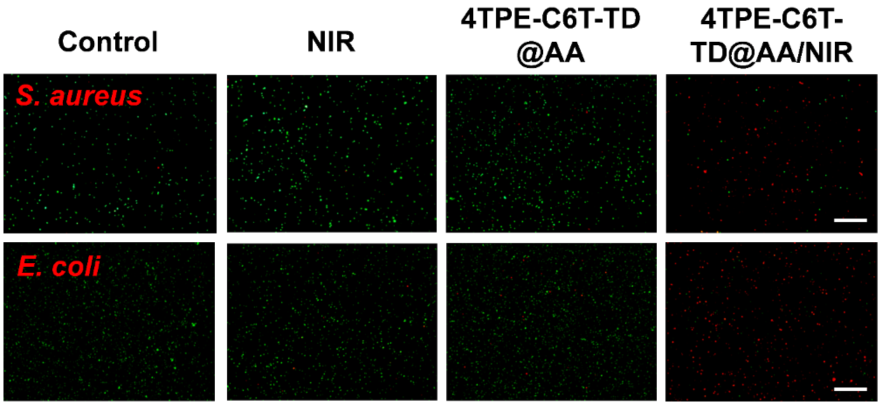


**Figure S30.** Live/Dead staining images of *S. aureus* and *E. coli*. Scale bar, 100 µm.

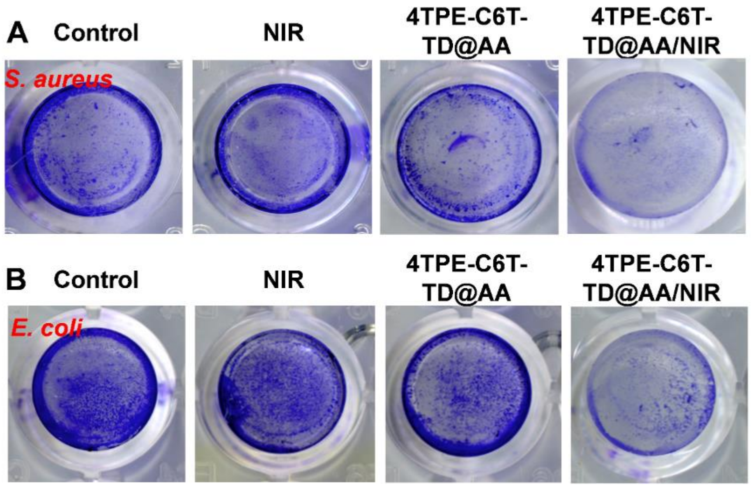


**Figure S31.** Crystal violet staining images of 24 h (A) *S. aureus* and (B) *E. coli* biofilms.

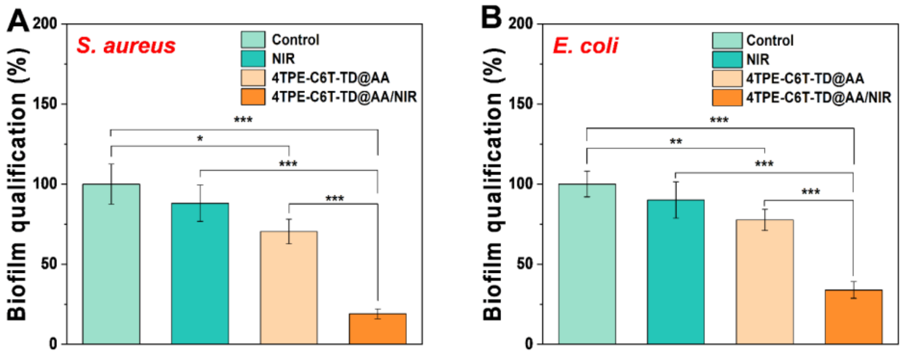


**Figure S32.** Biofilm qualification after different treatments of 24 h (A) *S. aureus* and (B) *E. coli* biofilms ($n$ = 3). Statistical analysis was performed using one-way ANOVA with Tukey's post-test. *$p < 0.05$, **$p < 0.01$ and ***$p < 0.001$.

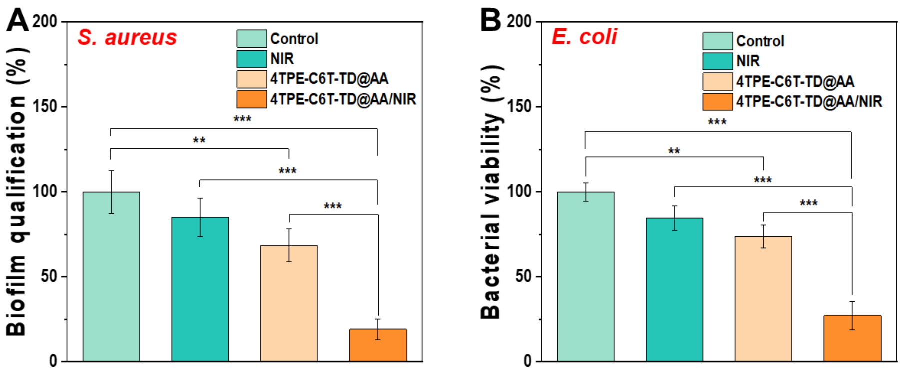


**Figure S33.** Biofilm qualification after different treatments of 7 days (A) *S. aureus* and (B) *E. coli* biofilms ($n$ = 3). Statistical analysis was performed using one-way ANOVA with Tukey's post-test. **$p < 0.01$ and ***$p < 0.001$.

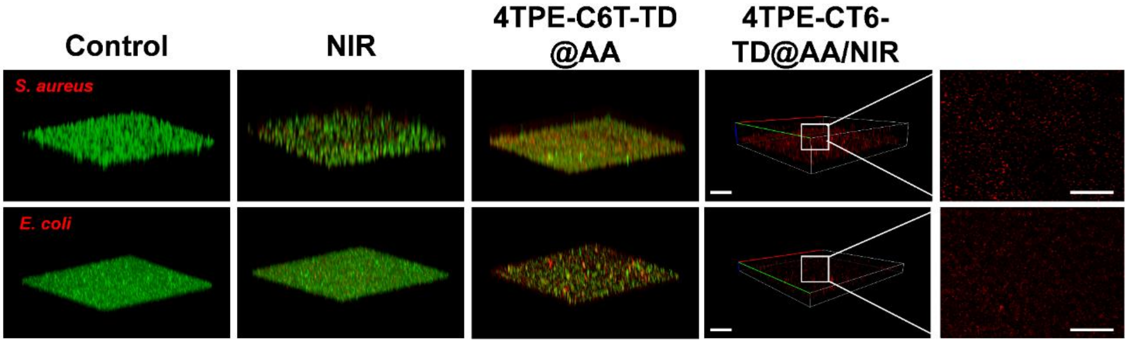


**Figure S34.** 3D confocal Live/Dead staining images of 24 h biofilms. Scale bar, 100 μm.

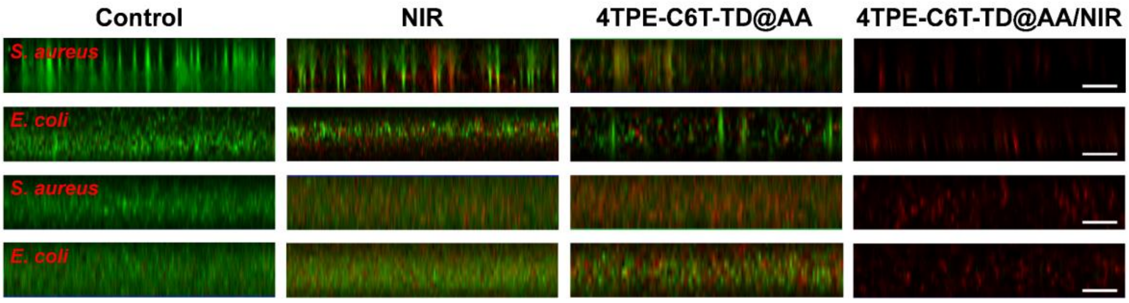


**Figure S35.** Cross section of Live/Dead staining of biofilm formed by *S. aureus* and *E. coli* for 24 h and 7 days. Scale bar, 20 μm.

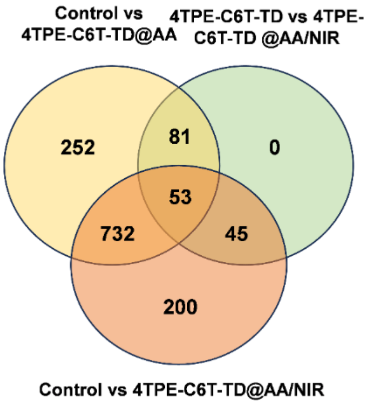


**Figure S36.** Venn Diagram of differentially expressed genes (DEGs) in *S. aureus* with different treatments.

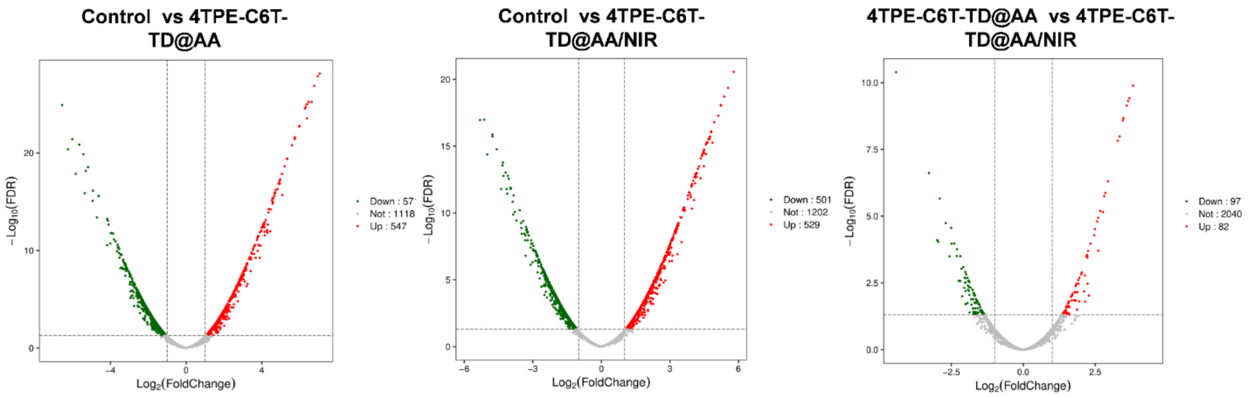


**Figure S37.** Volcano plot of DEGs in *S. aureus* with different treatments.

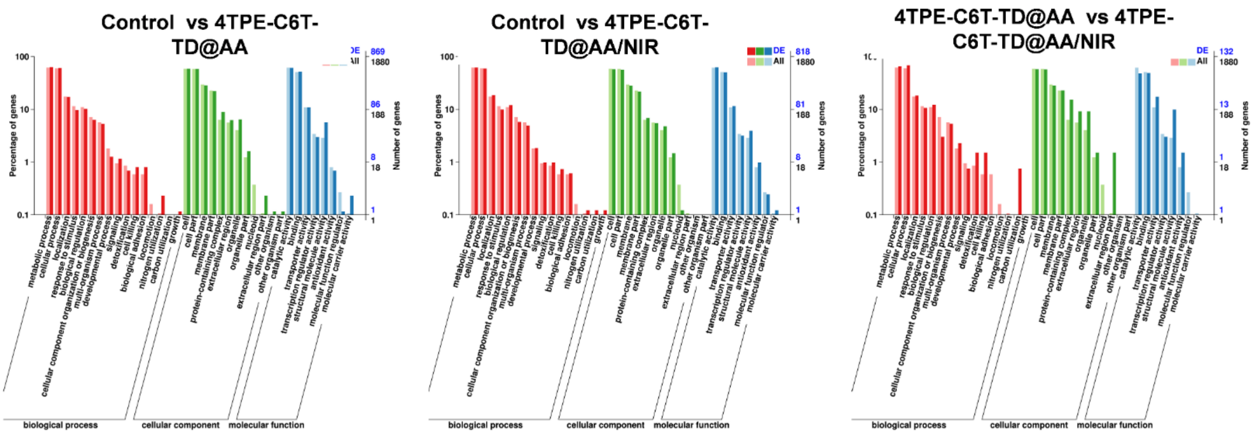


**Figure S38.** GO annotation analysis of DEGs in *S. aureus* with different treatments.

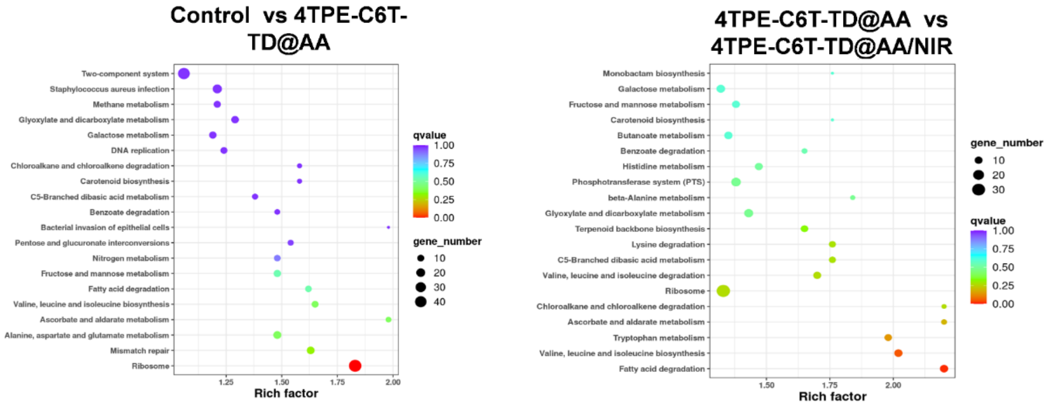


**Figure S39.** KEGG enrichment analysis of DEGs in *S. aureus* with different treatments.

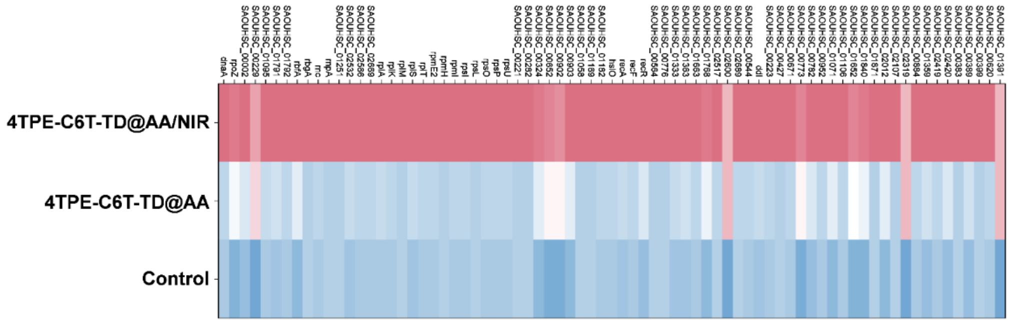


**Figure S40.** Heatmap of DEGs in *S. aureus* with different treatments.

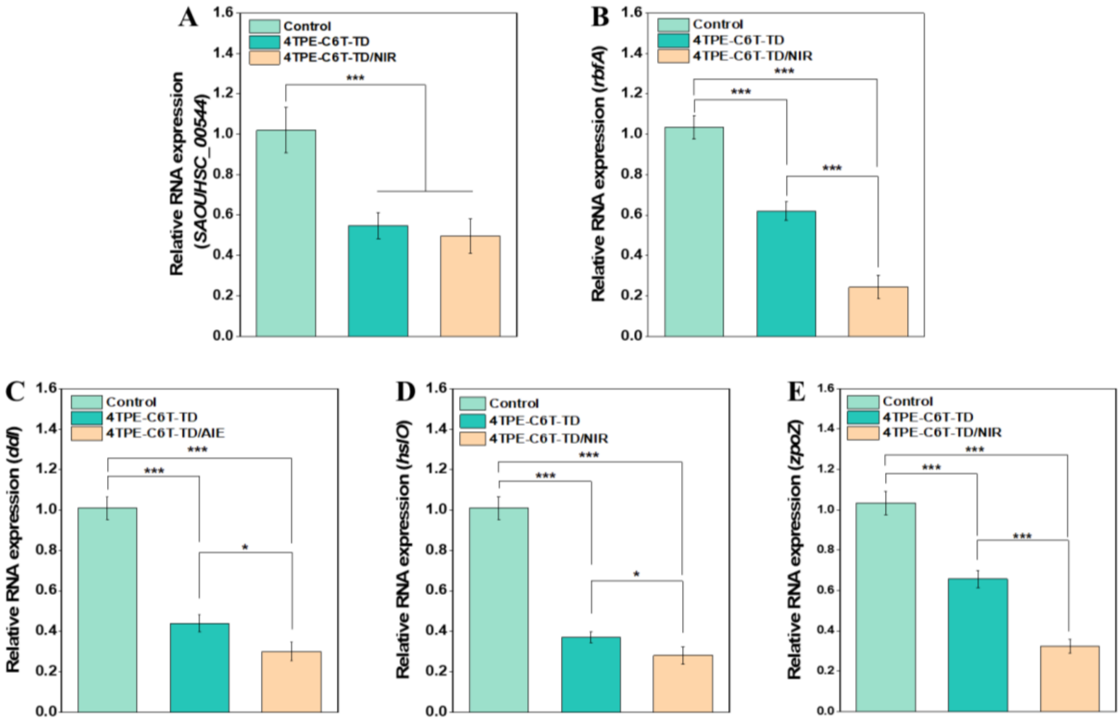


**Figure S41.** The mRNA expression levels of (A) *SAOUHSC_00544*, (B) *rbfA*, (C) *ddl*. (D) *hslO* and (E) *zpoZ* of *S. aureus* after different treatment ($n$ = 3). Statistical analysis was performed using one-way ANOVA with Tukey's post-test. *$p < 0.05$ and ***$p < 0.001$.

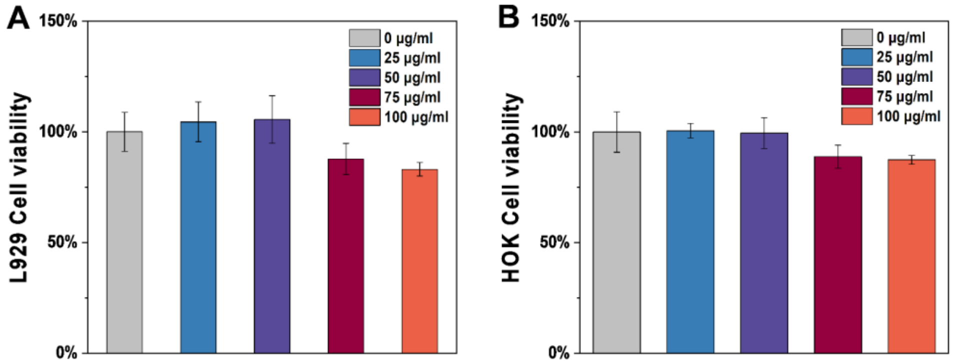


**Figure S42.** The survival rate of (A) L929 cells and (B) HOK cells co-cultured with 4TPE-C6T-TD@AA for 24 h ($n$ = 3). Statistical analysis was performed using one-way ANOVA with Tukey's post-test.

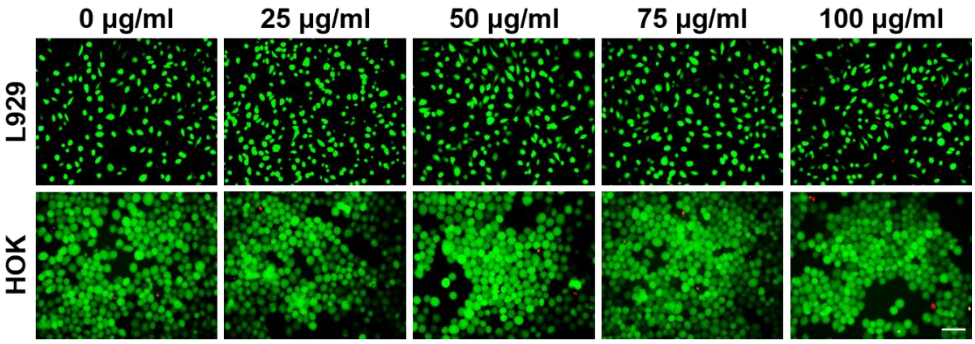


**Figure S43.** Live/Dead staining of A) L929 cells and B) HOK cells co-cultured with 4TPE-C6T-TD@AA for 24 h. Scale bar, 100 μm.

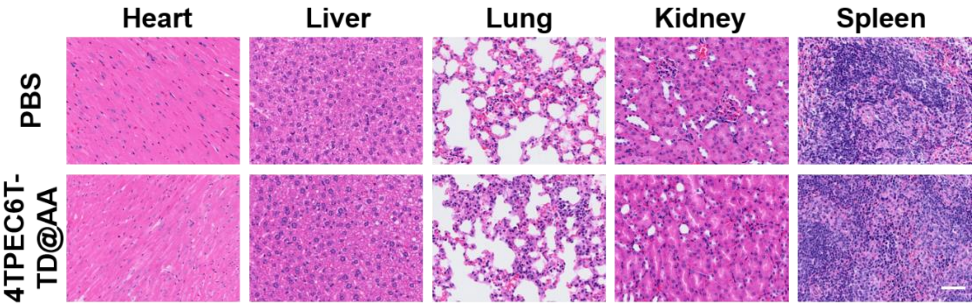


**Figure S44.** H&E staining images of hearts, livers, lungs, kidneys, and spleen in mice. Scale bar, 50 μm.

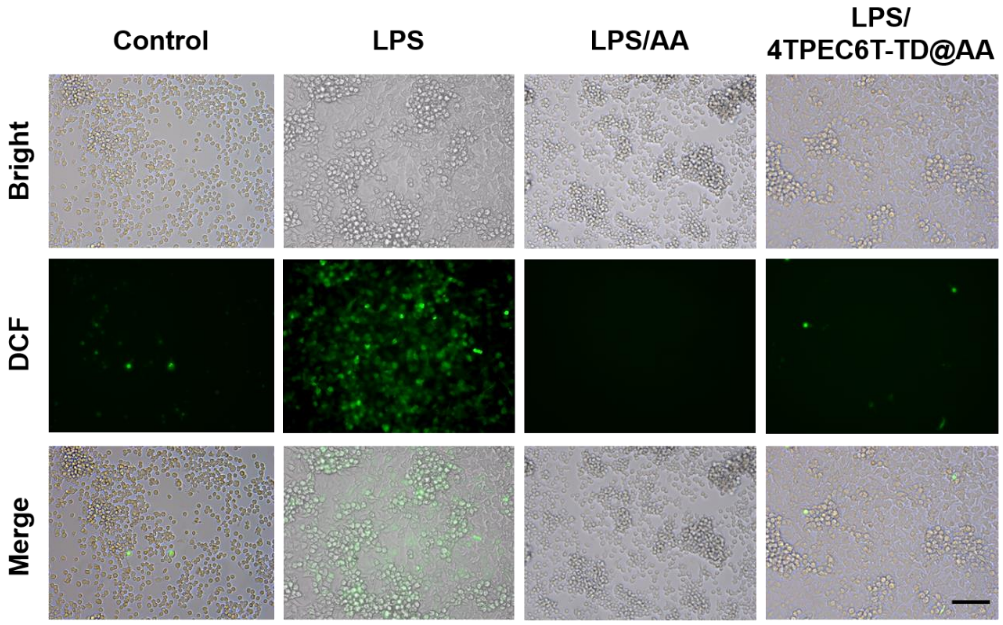


**Figure S45.** Fluorescent images of RAW264.7 macrophages under treatments of AA or 4TPE-C6T-TD@AA combined pretreatment of LPS. Scale bar, 100 μm.

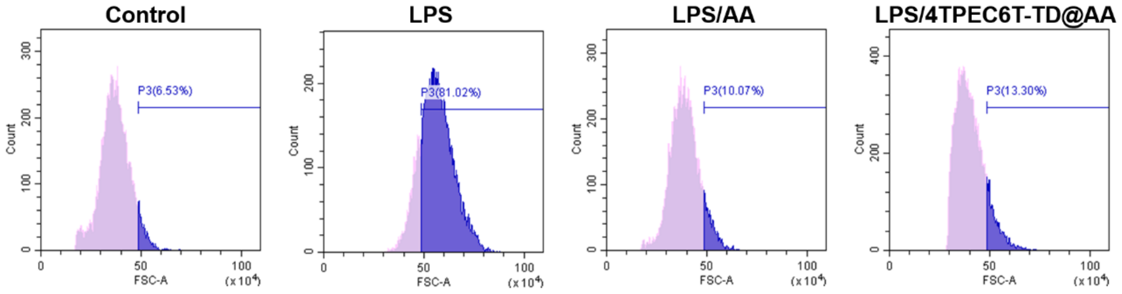


**Figure S46.** Flow cytometry analysis of of RAW264.7 macrophages under treatments of AA or 4TPE-C6T-TD@AA combined pretreatment of LPS.

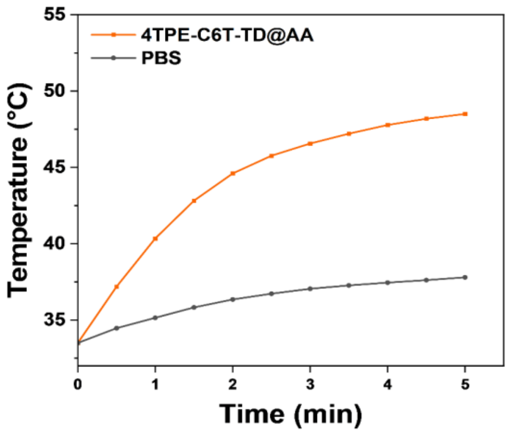


**Figure S47.** Temperature-time change curves of NIR and 4TPE-C6T-TD@AA/NIR groups.

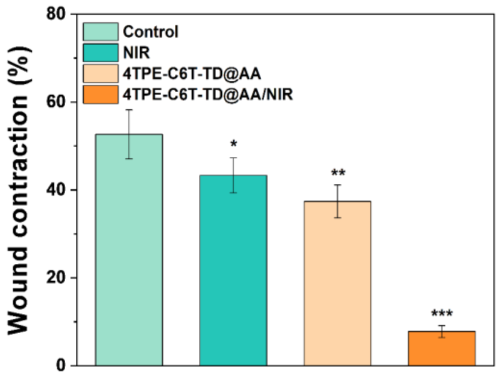


**Figure S48.** Postoperative and preoperative wound area ratio ($n$ = 3). Statistical analysis was performed using one-way ANOVA with Tukey's post-test. *$p < 0.05$, **$p < 0.01$ and ***$p < 0.001$.

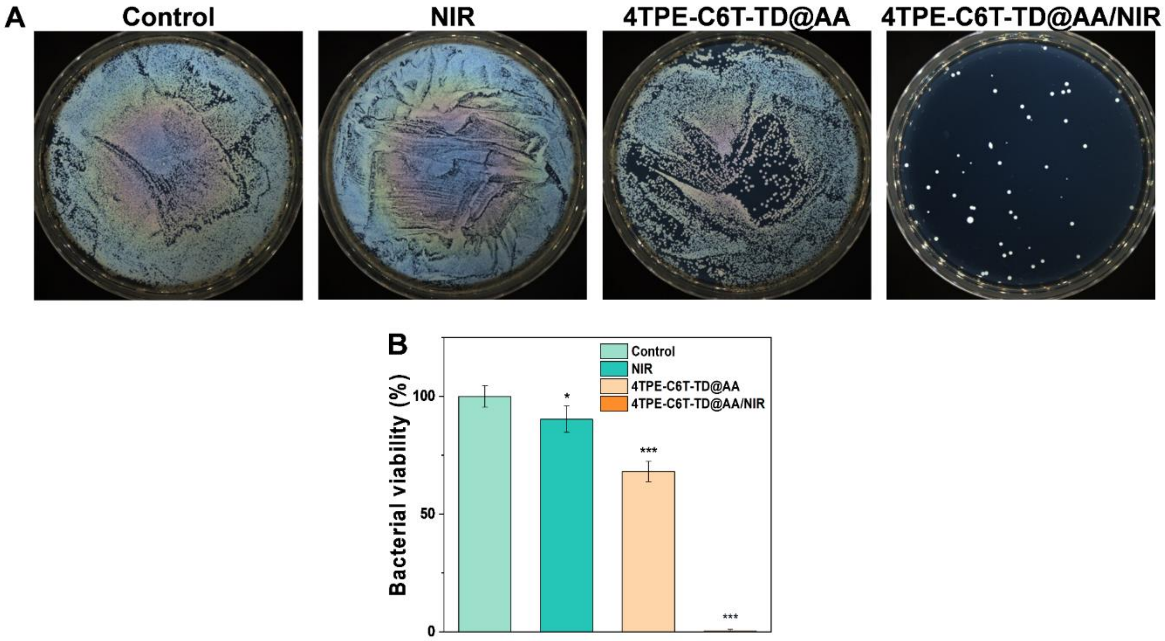


**Figure S49.** (A) Bacteria on the agar plates separated from wound tissue. (B) Quantitative analysis of bacterial survival rate ($n$ = 3). Statistical analysis was performed using one-way ANOVA with Tukey's post-test. *$p < 0.05$ and ***$p < 0.001$.

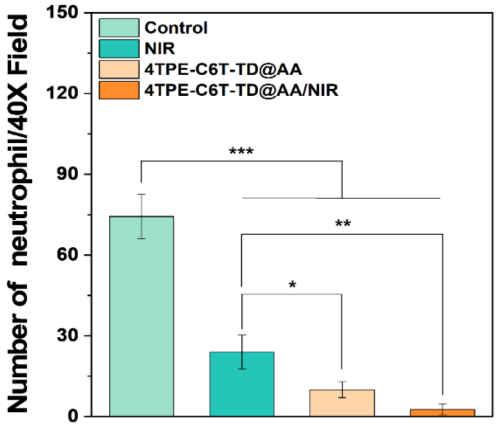


**Figure S50.** The number of neutrophils in H&E staining images ($n$ = 3). Statistical analysis was performed using one-way ANOVA with Tukey's post-test. *$p < 0.05$, **$p < 0.01$ and ***$p < 0.001$.

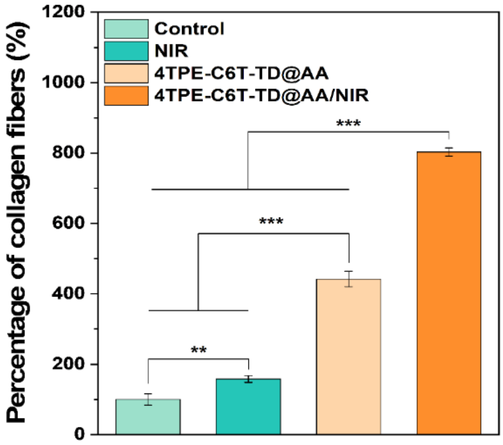


**Figure S51.** Collagen statistical data of Masson staining images ($n$ = 3). Statistical analysis was performed using one-way ANOVA with Tukey's post-test. **$p < 0.01$ and ***$p < 0.001$.

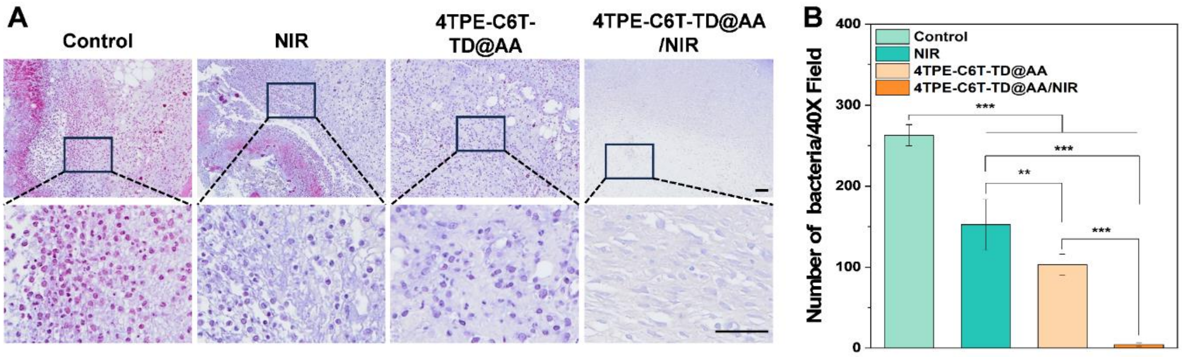


**Figure S52.** (A) Gram staining images of wound tissue in rats and (B) bacterial count ($n$ = 3). Scale bar, 50 μm. Statistical analysis was performed using one-way ANOVA with Tukey's post-

test. **$p < 0.01$ and ***$p < 0.001$.

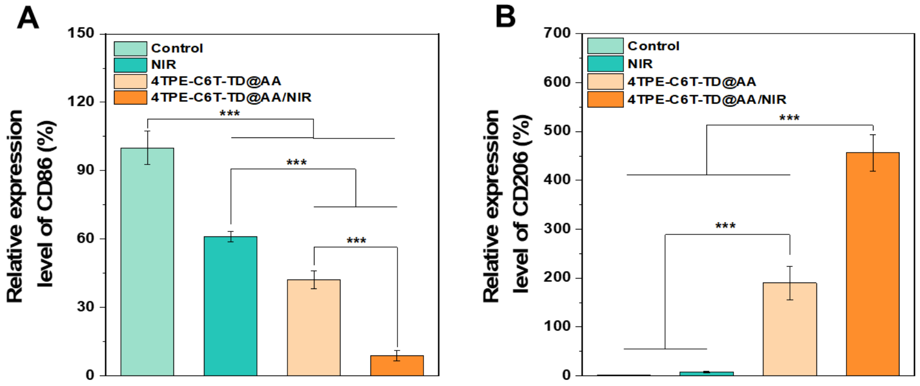


**Figure S53.** Statistics of positive expression of (A) CD86 and (B) CD206 in immunofluorescence staining images ($n = 3$). Statistical analysis was performed using one-way ANOVA with Tukey's post-test. ***$p < 0.001$.

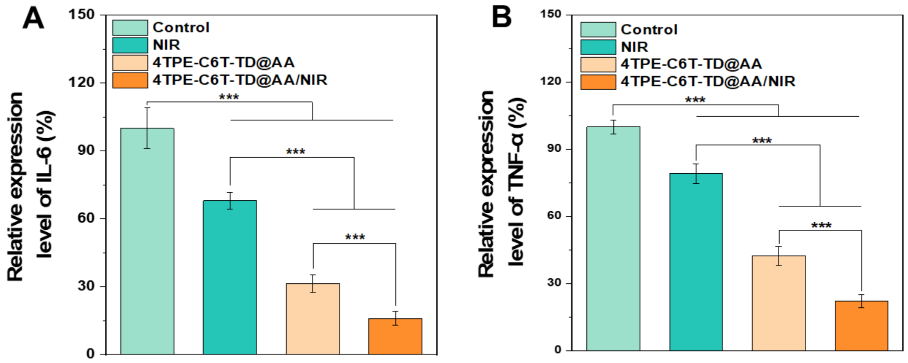


**Figure S54.** Statistics of positive expression of (A) IL-6 and (B) TNF-α in immunohistochemical staining images ($n = 3$). Statistical analysis was performed using one-way ANOVA with Tukey's post-test. ***$p < 0.001$.

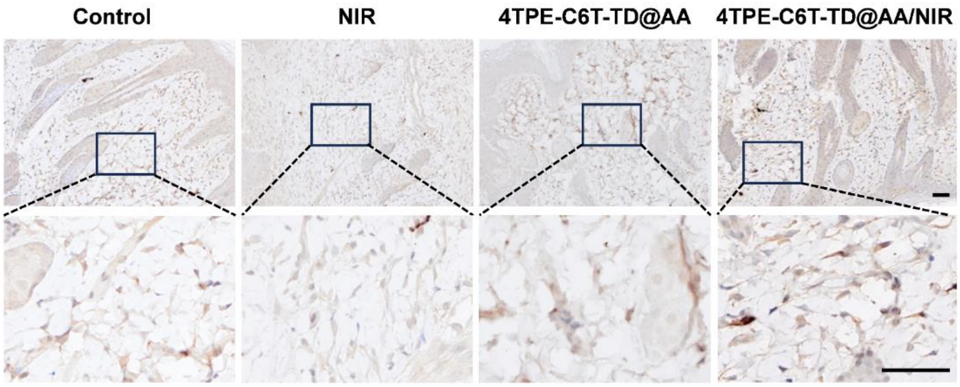


**Figure S55.** Immunohistochemical staining images of IL-10. Scale bar, 50 μm.

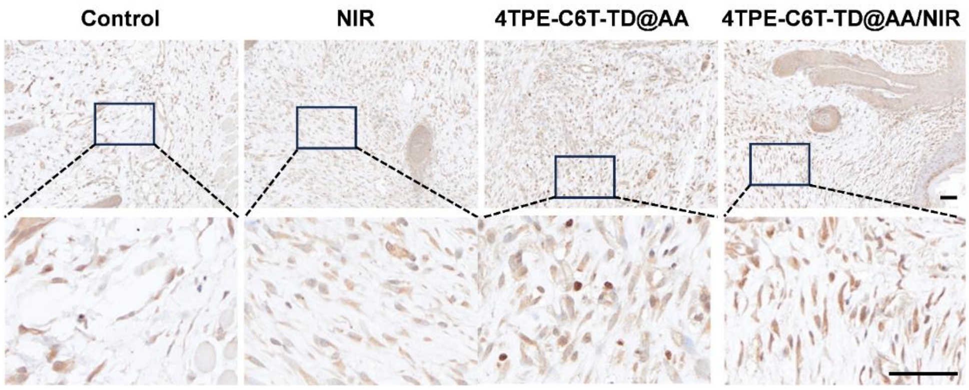


**Figure S56.** Immunohistochemical staining images of TGF-β. Scale bar, 50 μm.

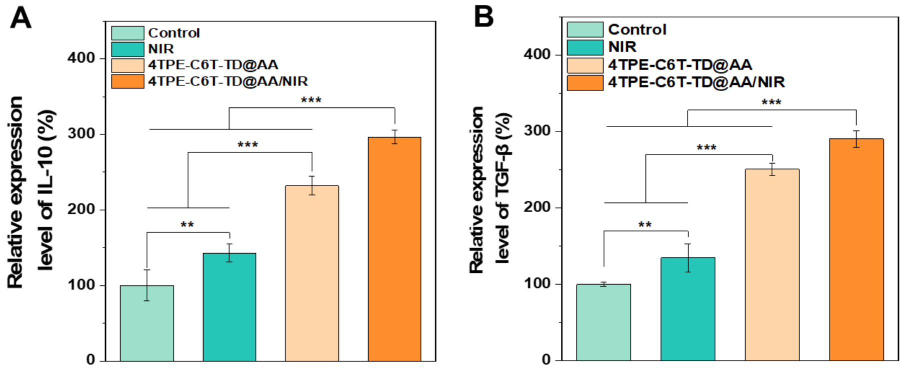


**Figure S57.** Statistics of positive expression of (A) IL-10 and (B) TGF-β in immunohistochemical staining images ($n$ = 3). Statistical analysis was performed using one-way ANOVA with Tukey's post-test. **$p < 0.01$, ***$p < 0.001$.

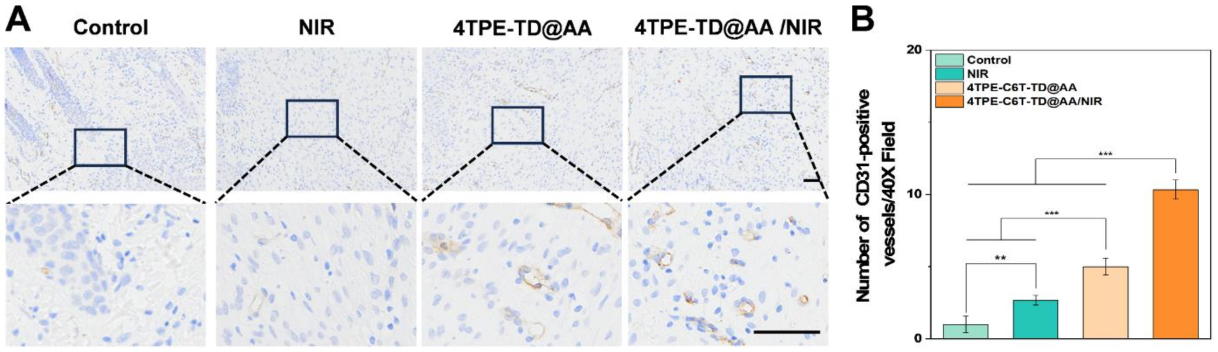


**Figure S58.** (A) Immunohistochemical staining images of CD31. Scale bar, 50 μm. (B) The number of CD31-positive vessels in Immunohistochemical staining images ($n$ = 3). Statistical analysis was performed using one-way ANOVA with Tukey's post-test. **$p < 0.01$ and ***$p < 0.001$.

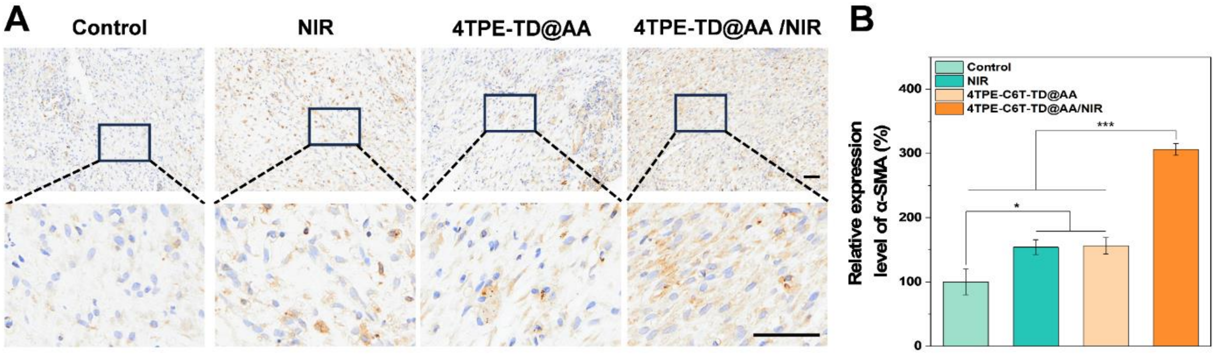


**Figure S59. (**A) Immunohistochemical staining images of α-SMA. Scale bar, 50 μm. (B) Statistics of positive expression of α-SMA in Immunohistochemical staining images ($n$ = 3). Statistical analysis was performed using one-way ANOVA with Tukey's post-test. **$p$ < 0.01 and ***$p$ < 0.001.

## Supporting tables

**Table S1.** Primer used for qRT-PCR of *S. aureus*.

| Gene | Forward primers | Reverse primers |
|---|---|---|
| *ddl* | TTTAGAAAATGGAGAGGCGCTTGAG | CGTGCCATCTTCACCATTAGGACCA |
| *rbfA* | TAAAGCAAAAGGCTTCATTAAGTCT | TCGATTGATTGATCATATTCATACA |
| *HslO* | ATGGGAAGAACAATGACAGCAA | GGATGGTCTACATAAGCACGCA |
| *rpoZ* | GCGTGAAATTGATGAACAACCTGAA | AACAGGGCGAATTTTACCGTCAGCA |
| *SAOUHSC_ 00544* | ATGCAATCACCACAAAACGC | GTAGTTGAAACATCTTTTGCTTG |
| *16s* | TGCGGGACTTAACCCAACA | TGGAGCATGTGGTTTAATTCGA |

**Table S2.** Primer used for qRT-PCR of immune factors.

| Gene | Forward primers | Reverse primers |
|---|---|---|
| *IL-1β* | TGGAGAGTGTGGATCCCAAG | GGTGCTGATGTACCAGTTGG |
| *IL-6* | CTGAACTTCGGGGTGATCGG | GGCTTGTCACTCGAATTTTGAGA |
| *CD206* | AGACGAAATCCCTGCTACTG | CACCCATTCGAAGGCATTC |
| *IL-10* | GAGAAGCATGGCCCAGAAATC | GAGAAATCGATGACAGCGCC |

## REFERENCES

1. Meyer CT, Lynch GK, Stamo DF *et al*. A high-throughput and low-waste viability assay for microbes. *Nat Microbiol* 2023; **8**: 2304-2314.
